# A split luciferase biosensing platform for detection and imaging of chromatin loops in individual live cells

**DOI:** 10.1093/nar/gkaf1324

**Published:** 2025-12-03

**Authors:** Nicholas G Heath, J Antonio Gomez, Sean P McGinty, Henriette O’Geen, David J Segal

**Affiliations:** Genome Center, University of California, Davis, CA 95616, United States; Genome Center, University of California, Davis, CA 95616, United States; Natural Science Division, Pepperdine University, Malibu, CA 90263, United States; Genome Center, University of California, Davis, CA 95616, United States; Genome Center, University of California, Davis, CA 95616, United States; Genome Center, University of California, Davis, CA 95616, United States; Department of Biochemistry and Molecular Medicine, University of California, Davis, CA 95616, United States

## Abstract

Eukaryotic cells regulate higher-order chromatin architecture, gene expression, and gene recombination via compaction of the genome into chromatin loops and topologically associating domains (TADs). While chromatin architecture has been thoroughly characterized for many eukaryotic genomes using cell-destructive techniques such as 3C-based methods, live-cell biosensing tools that can probe three-dimensional chromatin contacts in real-time are lacking. Using a dual dCas9 DNA biosensor based on a split NanoLuc luciferase reporter, we directly detected chromatin loops in live cells using luminescence quantification in a luminometer. We observed signal-to-background ratios of up to 10-fold. In addition, we directly visualized chromatin looping at the *MYC* TAD in live cells using high-resolution, low-light live-cell imaging. Our biosensing platform therefore provides a useful methodology for live-cell, real-time detection of known or novel loops and for monitoring looping dynamics upon alterations in cell state.

## Graphical Abstract

**Graphical Abstract:**
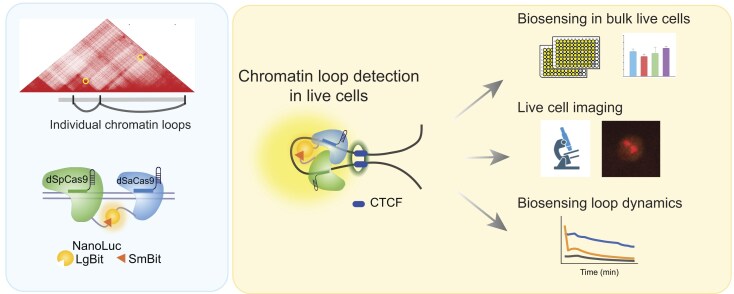

## Introduction

Changes in chromatin structure are implicated in disease pathogenesis, making chromatin loops a biologically significant target for detection and tracking in live cells. Although probes based on zinc finger proteins, TALE, and CRISPR–Cas9 combined with fluorescent reporters have recently been developed to track chromatin dynamics in live cells using super-resolution microscopy and other microscopy-based techniques [1–8], these tools have several limitations, including high autofluorescent background, cellular phototoxicity, and rapid photobleaching of fluorophores and other fluorescent reporters, requiring a large number of target–bound probe complexes to reliably distinguish the target-specific foci from the background [9]. In addition, cell-destructive methods such as ChIA-PET, Hi-C, and HiChIP provide a time- and population-averaged representation of the landscape of chromatin contacts in cells, revealing a retrospective analysis of changes in chromatin interactions [10–12]. While these methods have achieved impressive kilobase resolution for chromatin interactions, sensitive detection methods to probe point interactions at even higher resolution are lacking [13, 14]. A powerful approach to fill this gap would be to harness bioluminescence-based DNA biosensors, enabling quantification of three-dimensional chromatin contacts at base-pair resolution, noninvasively and in real-time, in individual live cells and across populations of live cells.

Chromatin loops are created by a loop extrusion process mediated by cohesin, a ring-shaped DNA-entrapping adenosine triphosphatase (ATPase) complex, and CCCTC-binding factor (CTCF). Recent studies have shown that formation of topologically associating domains (TADs) is highly dependent on individual CTCF site DNA orientation, with tandem and convergent CTCF sites typically forming boundaries within TADs and divergent CTCF sites forming boundaries between TADs [15, 16]. Chromatin loops form via cohesin initially extruding a loop of DNA bidirectionally until it forms a complex with CTCF bound to its cognate binding site on the DNA [17–20]. Cohesin continues to extrude a loop through its ring-shaped structure by sliding an opposing DNA sequence toward the first bound CTCF until encountering a second CTCF bound to the other DNA strand in an appropriate orientation, where CTCF proteins homodimerize to form a loop anchor [17–20]. We hypothesized that the loop extrusion mechanism might create a relatively short bridge or anchor region at the base of a chromatin loop, enabling the two components of a DNA-binding split biosensor to be brought close enough together to produce a detectable signal over background at targetable sites around a cohesin-CTCF homodimer-anchored loop.

Here, we describe a live-cell NanoLuc-based DNA biosensor composed of LgBiT-d*Sa*Cas9 and d*Sp*Cas9-SmBiT fusion proteins that bridge the loop anchor regions. We demonstrate detection of insulating chromatin loops at TAD boundaries at up to 10.5-fold above background and intra-TAD loops at up to 6.1-fold above background in common cancer cell lines using high-resolution, low-light live-cell imaging. We show that when one of the two looping loci containing one of the CTCF binding sites is deleted, the on-target biosensing signal decreases to near-background levels. These proof-of-concept results demonstrate the roles of cohesin and CTCF in the output of luminescence from our biosensor, providing a reliable method for the detection of cohesin and CTCF homodimer-anchored chromatin loops in live cells.

## Materials and methods

### Construction of directional d*Sp*Cas9-NanoBiT, NanoBiT-d*Sp*Cas9, d*Sa*Cas9-NanoBiT, and NanoBiT- d*Sa*Cas9 fusion proteins

The directional fusion constructs containing the LgBiT and SmBiT of NanoLuc luciferase (Promega Corporation) fused to catalytically inactive Cas9 enzymes from *Streptococcus pyogenes* and *Staphylococcus aureus* were generated using the Gibson Assembly method (New England Biolabs). For d*Sp*Cas9 fusion constructs, we used an improved version of the pCDNA3-d*Sp*Cas9 vector (Addgene, #100091) containing two nuclear localization signals, an N-terminal 3× Flag epitope tag, and [(GGS)5] flexible linker sequences, as well as two separate multiple cloning sites at the N- and C-termini of dCas9, and cloned HaloTag, NanoLuc, LgBiT, and SmBiT onto the N- and C-termini of d*Sp*Cas9 using two separate N- and C-terminal multiple cloning sites as described in our previously published study [21]. All sequences are listed in Supplementary data (Supplementary Note S2). HaloTag, NanoLuc, LgBiT, and SmBiT inserts were ordered as double-stranded DNA fragments or gBlocks Gene Fragments (Azenta Life Sciences and Integrated DNA Technologies) containing ~45 bp homologous sequences with the doubly digested d*Sp*Cas9 vectors for Gibson cloning. NLuc-d*Sp*Cas9 fusion construct was created in our previously published study [21] using overlap extension PCR on LgBiT-d*Sp*Cas9 and SmBiT-d*Sp*Cas9 gBlocks to directionally splice the sequences, followed by the Gibson Assembly method again using the N-terminal double-digested d*Sp*Cas9 vector.

For d*Sa*Cas9 fusion constructs, we replaced d*Sp*Cas9 in pCDNA-KRAB-d*Sp*Cas9 (Addgene, #112195) with the d*Sa*Cas9 coding sequence. The resulting pCDNA-KRAB-d*Sa*Cas9 vector contains two nuclear localization signals, an N-terminal 3× FLAG epitope tag, [(GGS)5] flexible linker sequences, and two separate multiple cloning sites at the N- and C-termini of d*Sa*Cas9. We cloned HaloTag, NanoLuc luciferase, LgBiT, and SmBiT onto the N- and C-termini of d*Sa*Cas9 using two separate N- and C-terminal multiple cloning sites in the pCDNA-KRAB-d*Sa*Cas9 vector. The necessary vector backbone was produced with the KRAB domain removed for subsequent N-terminal Gibson Assembly reactions. For C-terminal constructs, this vector was further processed to create an appropriate vector backbone with resealed N-terminal multiple cloning site. Two more short oligos created a short 9-nucleotide adapter sequence, adding 3 amino acids between KpnI and FseI for C-terminal constructs, resulting in a 16-amino-acid flexible linker between the 3× FLAG epitope and the N-terminus of d*Sa*Cas9. Then, an NheI/NotI linearized vector backbone was used for subsequent C-terminal Gibson Assembly reactions. HaloTag, NanoLuc, LgBiT, and SmBiT inserts were ordered as double-stranded DNA fragments or gBlocks Gene Fragments (Azenta Life Sciences and Integrated DNA Technologies) containing ~45 bp sequences homologous to the double-digested dCas9 vectors (see Supplementary data).

### Construction of sgRNA expression cassettes and plasmids

The dSpCas9 sgRNA cloning vector (Addgene, plasmid #41824) was linearized using AflII, and sgRNA sequences were inserted using Gibson Assembly [22]. For d*Sa*Cas9 sgRNA cloning, the *Sa*gRNA cassette was synthesized (see Supplementary Note S3). The 19-bp d*Sp*Cas9 and 21-bp d*Sa*Cas9 sgRNA sequences that target unique (non-repetitive) *MUC4* DNA sequences (see Supplementary Note S4) were selected using a combination of design tools including CHOPCHOP, CRISPRscan, CRISPick, CRISPOR, and the UCSC Genome Browser to maximize specificity for the on-target loci. All vectors were sequenced to confirm exact sequences were present.

### Chromatin loop biosensing using the tecan spark multimode plate reader

For chromatin loop biosensing assays using the Tecan Spark Multimode Plate Reader, HEK293T, HCT116, and K562 cells were originally purchased from ATCC (CCL-247 and CCL-243, respectively) and maintained in DMEM, McCoy’s 5A, and RPMI 1640 (Thermo Fisher Scientific), respectively. Growth media were supplemented with 10% fetal bovine serum (FBS) and 1× penicillin/streptomycin, and cells were maintained at 37°C under 5% CO_2_. Low-passage HCT116 cells were seeded at 2 × 10^4^ cells per well in 96-well opaque white translucent bottom assay plates (Thermo Fisher Scientific) ~20 h prior to transfection. One hundred nanograms of total DNA was transiently transfected in each well using the Lipofectamine 3000 protocol (Thermo Fisher Scientific), consisting of 29 ng (5 fmol) LgBiT-d*Sa*Cas9 fusion construct, 31 ng d*Sp*Cas9-SmBiT fusion construct (5 fmol), 13 ng d*Sp*Cas9 sgRNA expression plasmid (5 fmol), 1.41 ng d*Sa*Cas9 sgRNA expression cassette (5 fmol), and 25.59 ng pMAX-GFP plasmid. Four biological and technical replicates were included for each *cis*-interacting loop anchor sgRNA pair. HCT116 cells were typically transfected at 70%–85% efficiency. Low-passage K562 cells were counted, and 4 × 10^5^ cells were washed once with 1× DPBS and resuspended in 20 µl resuspension buffer R (Thermo Fisher Scientific). Five hundred nanograms of total DNA was transiently transfected via electroporation (1450 V, 10 ms pulse width, 3 pulses) using the Neon Transfection System (Thermo Fisher Scientific), consisting of 180 ng (30 fmol) LgBiT-d*Sa*Cas9 fusion construct, 192 ng d*Sp*Cas9-SmBiT fusion construct (30 fmol), 81 ng d*Sp*Cas9 sgRNA expression plasmid (30 fmol), 8.75 ng d*Sa*Cas9 sgRNA expression cassette (30 fmol), and 38.25 ng pMAX-GFP plasmid. Four biological and technical replicate electroporations were included for each *cis*-interacting loop anchor sgRNA pair using two 10 µl Neon tips (Thermo Fisher Scientific) and plated in 24-well cell culture plates (Olympus) with 500 µl RPMI 1640 + 10% FBS without antibiotics. K562 cells were typically transfected with >95% efficiency. Twenty-four hours post-electroporation, cells were resuspended in 400 µl fresh RPMI 1640 + 10% FBS without antibiotics, and 100 µl were added to a 96-well opaque white translucent bottom assay plate (Thermo Fisher Scientific). All genomic target site sequences for d*Sp*Cas9 and d*Sa*Cas9 sgRNAs are listed in Supplementary Note S5. For each cell line, 24 h post-transfection, Nano-Glo Live Cell Substrate (furimazine) was diluted 1:20 in Nano-Glo LCS Dilution Buffer (Promega Corporation), and 25 µl reconstituted Nano-Glo Live Cell Substrate was added to each well. For single time point assays, 10 min after addition of the substrate, luminescence (total photon counts, 1500 ms integration time) was recorded using the Tecan Spark with a band-pass (BP) filter centered at the peak emission wavelength of 460 nm with a band-pass range of 200 nm from 360 to 560 nm. For time-course biosensing assays, 10 min after addition of luminescent substrate, wild-type HCT116 or HCT116-RAD21-mAC OsTIR1(F74G) (HCT116-RAD21-mAC for short) cells were treated with either DMSO (untreated) or 1 µM auxin (5-Ph-IAA), marking time point 0. Then, luminescence (total photon counts, 1500 ms integration time) was read every 5 min for 85–105 min starting at time point 0. For control “non-loop” sgRNAs, a pair of sgRNAs targeting noninteracting genomic regions was transfected (*PALB2* d*Sp*Cas9 sgRNA 4 from our previous study and *MYC* promoter d*Sa*Cas9 sgRNA d) [21]. For all transfections, in conditions where no sgRNAs were transfected, 14.4 ng of inert pUC19 vector was added to the transfection mix. For positive control NanoLuc-d*Sp*Cas9 and negative control LgBiT-dSaCas9 alone conditions, 32.4 ng inert pUC19 vector was added to the transfection mix. For negative control SmBiT-d*Sp*Cas9 alone condition, 42 ng of inert pUC19 vector was added to the transfection mix.

### Chromatin loop biosensing using low-light microscopy on an Andor Dragonfly 200 Spinning Disc Confocal Microscope and image processing

For microscopy sessions on the Andor Dragonfly 200 Multi-modal Confocal System, low-passage HCT116 cells were plated in 35 mm diameter CELLview 4-quadrant glass bottom imaging dishes (Greiner Bio-One) and transfected identically to experiments using the Tecan Spark Multimode Plate Reader, except that Lipofectamine 3000 (Thermo Fisher Scientific) transfections were scaled up for 24-well plate growth area (1.9 cm^2^). Briefly, 2 × 10^5^ or 2.5 × 10^5^ cells were plated in each quadrant of the imaging dishes, and 500 ng total DNA was transfected in each quadrant, consisting of 180 ng LgBiT-d*Sa*Cas9 fusion construct (30 fmol), 192 ng d*Sp*Cas9-SmBiT fusion construct (30 fmol), 81 ng d*Sp*Cas9 sgRNA expression plasmid (30 fmol), 8.75 ng d*Sa*Cas9 sgRNA expression cassette (30 fmol), and 38.25 ng pMAX-GFP plasmid. Four biological and technical replicate quadrants were included for each *cis*-interacting loop anchor sgRNA pair or control condition transfected. Thirty-five hours post-transfection, Nano-Glo Live Cell Substrate (furimazine) was diluted 1:20 in Nano-Glo LCS Dilution Buffer (Promega Corporation), and 200 µl reconstituted Nano-Glo Live Cell Substrate was added to 300 µl McCoy’s 5A Medium (Thermo Fisher Scientific) in each quadrant of the imaging dishes. Fifteen minutes after the addition of the luminescent substrate, GFP fluorescence and NanoLuc luminescence were measured on the Andor Dragonfly Spinning Disc Confocal Microscope. An optimized luminescence imaging protocol was developed for use on the Andor Dragonfly 200 equipped with the Andor iXon Ultra 888 EMCCD camera, in which cells were placed in an imaging chamber with temperature control at 37°C and all major exogenous light sources in the room were blocked. Using the widefield imaging modality, NanoLuc luminescence was subsequently imaged at 63× magnification with oil immersion using the DAPI channel with 405 nm laser set to minimum intensity (0.002%), shutter turned off, exposure time set to 15 s, and EM gain set to 300. We also changed settings in the Andor image acquisition software to 1× magnification under “Confocal Limit” and to 100% pass under “Image Splitter.” GFP fluorescence was imaged using an exposure time of 2 ms and EM gain of 300.

Minimal post-processing was applied to better visualize the images. Raw 16-bit grayscale GFP images were recolored using the “green” look up table (LUT) in Fiji and brightness was reduced to approximately 50% of maximum for each image. Raw 16-bit grayscale NanoLuc luminescence images were recolored using the “red” LUT, brightness was increased to 100%, and contrast was increased to approximately 50% of maximum for each image. To merge GFP fluorescence and NanoLuc luminescence images, we directly merged color channels in Fiji. The WEKA Segmentation package [23] in Fiji was then used to segment cell nuclei using 25 ROI traces of the nuclear luminescence signals and 25 ROI traces of the background outside of cells as a training data set for nuclear boundaries. This trained WEKA segmentation model was then applied to each NanoLuc image to determine boundaries of nuclei. Each 8-bit segmented image outputted from the WEKA model was binarized using the “auto-threshold” function, and the “analyze particles” function was applied to create ROIs for each nuclear area. These ROIs were then overlaid from the ROI manager onto unprocessed images for quantification. Then, the mean intensity (equivalent to integrated intensity of each nucleus divided by area of each nucleus) after 15 s of total light collection was calculated and recorded for each segmented nuclear area using Fiji. Any cells that were positive for GFP fluorescence but negative for NanoLuc luminescence were omitted from final statistical analysis.

### 4C-seq

4C-seq was conducted as described previously [24–27]. For each replicate, ~5 million cells were crosslinked in 2% formaldehyde and 10% FBS in PBS for 10 min while rotating at room temperature. Glycine was added to a final concentration of 0.125 M to quench crosslinking reaction, and cells were centrifuged at 500 x *g* for 5 min. Cells were washed twice with PBS, transferred to an Eppendorf tube, and lysed on ice for 20 min with 1 ml lysis buffer [50 mM Tris–HCl (pH 7.5), 10 mM NaCl, 5 mM EDTA, 0.5% NP-40, and 1% Triton X-100] supplemented with 1x protease inhibitors (Roche). Cells were then washed once with 450 µl of 1.2x DpnII buffer and resuspended in 500 µl 1.2x DpnII buffer and 15 µl of 10% SDS (final: 0.3% SDS) and incubated in a thermoshaker at 37°C for 1 h at 750 RPM. Then, 75 µl of 20% Triton X-100 (final: 2.5% Triton-X-100) was added, nuclear aggregates were resuspended with a pipet, and tubes were incubated at 37°C for 1 h while shaking at 750 RPM. A 5 µl aliquot of undigested control was stored, and nuclei were incubated overnight with 250 U of DpnII restriction enzyme (New England Biolabs). A fresh 250 U of DpnII was added the following morning, and samples were digested for an additional 3 h. After this, digestion was checked for completion by running 5 μl of sample on a 0.6% agarose gel next to undigested lysates. DpnII was inactivated by incubating the reaction tubes for 20 min at 65°C, and a proximity ligation reaction was performed in a 7 ml volume with 3200 U of T4 DNA ligase (New England Biolabs) for 16 h at room temperature, followed by an overnight period at 16°C and finally a 500 U T4 DNA ligase spike-in and another 36 h incubation at room temperature. After this, ligation was checked for completion by running 5 μl of sample on a 0.6% agarose gel next to the digested control. Cross-links were reversed at 65°C overnight after adding 300 μg proteinase K. Samples were then treated with 300 μg RNase A for 30 min at 37°C, and DNA was purified using MagMax DNA binding beads (Thermo Fisher Scientific) and a magnetic bead-based protocol described in a previous reference [26]. A second restriction digest was performed overnight at 37°C while shaking at 500 RPM in a 500 μl reaction with 200 U of NlaIII (New England Biolabs). After this, digestion was checked for completion by running 5 μl of sample on a 0.6% agarose gel next to the samples from the first ligation. NlaIII was inactivated at 65°C for 25 min, and a proximity ligation reaction (5 ng/μl DNA concentration) was performed in a 5 ml volume with 2000 U of T4 DNA ligase for 16 h at room temperature, followed by an overnight period at 16°C and finally a 400 U T4 DNA ligase spike-in and another 36 h incubation at room temperature. After ligation, DNA was again purified using MagMax DNA binding beads (Thermo Fisher Scientific).

To generate the 4C–seq library, several viewpoint-specific inverse PCR primer sets targeting the *MYC* promoter conserved CTCF binding site (*see*  Supplementary Note S6 for viewpoint and primer sequences) were compared for efficiency, and the primer pair that produced the strongest product was selected for the 1st round PCR in library preparation. Two hundred nanograms of prepared 4C template was amplified with 16 PCR cycles in four separate 50 μl reactions using Q5 Hot Start High-Fidelity DNA Polymerase (New England Biolabs). Next, these four reactions were mixed, and 50 μl of 1st round PCR product was purified using the ChargeSwitch PCR Clean-Up Kit (Thermo Fisher Scientific) to remove primer dimers. Five microliters of purified 1st round PCR product was used as a template in a 2nd round of PCR for 20 cycles in a 50 μl reaction using Q5 Hot Start High-Fidelity DNA Polymerase (New England Biolabs) to add Illumina P5/P7 flow cell adapters, Illumina sequencing primer sites, and unique 6-bp indexes to all replicates. These 2nd round reactions were then purified with the QIAquick PCR purification kit (QIAGEN); the purity was measured with a Nanodrop 2000 Spectrophotometer (Thermo Fisher Scientific); the concentration was more accurately determined using the Qubit dsDNA HS Assay (Thermo Fisher Scientific); and all replicates were run on the Bioanalyzer 2100 (Agilent) to check for any remaining primer dimers and determine average library size. After final quality control and Element library circularization, the 4C-seq libraries were sequenced on the Element Biosciences AVITI for 150 bases in paired-end read mode at 300 cycles. Demultiplexed fastq files were trimmed from the 5′ end to the start of the DpnII motif and mapped to the reference genome (hg19) using Bowtie 2.5.1. No mismatches were tolerated, and unmapped reads were removed. The reference genome was then digested *in silico*, and the fragment ends were mapped against the reference genome. Read counts were normalized (max total sum of mapped reads = 1 million) and smoothed (running mean window size = 21), and processed data files were generated. 4C peaks were called using the peakC R package (https://github.com/deWitLab/peakC).

### Region capture micro-C analysis

RCMC (region capture micro-C) data for HCT116 and K562 were downloaded at 50-bp bin sizes from https://zenodo.org/records/15303879 [28]. The *MYC* TAD locus (hg38 chr8:124 988 367–129 795 630) is among the capture regions. mcool files were converted to hic files using *hictk convert* v2.1.4 (https://github.com/paulsengroup/hictk, [29]). Raw read counts were normalized using vanilla coverage (VC) with *hictk balance*. VC-normalized contact matrices were visualized using the UCSC Genome Browser at 1.6-kbp resolution. VC scores were extracted for 200-bp bins overlapping gRNAs in each gRNA pair using *bedtools intersect* v2.26.0. We then performed linear regression analysis between the VC contact score and the SBR measure for each gRNA pair, calculating correlation coefficients and significance in R using *stats* v4.4.1 and *ggplot2* v3.5.1 (Supplementary Fig. S8).

### Statistical testing

Two-tailed Student’s *t*-tests for signal-to-background ratio (SBR) analyses and Pearson’s correlation analyses were conducted in Microsoft Excel 2016. Statistics shown in all box-and-whisker and time-series plots were computed using R (version 4.3.0).

## Results

### Engineering a dCas9-based dual-species biosensor

**Figure 1.**
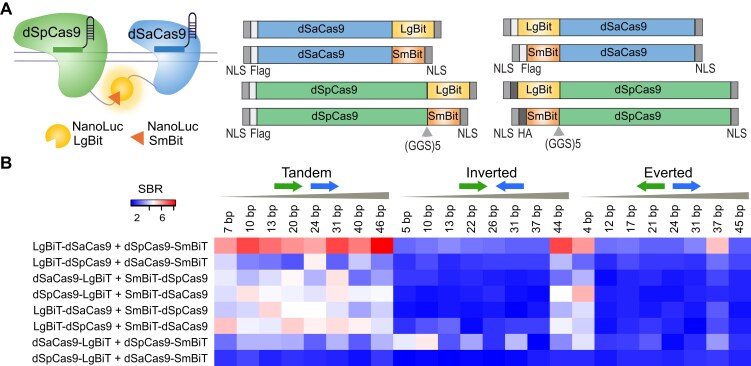
Engineering a dCas9-based dual-species biosensor. (**A**) Cartoon of the C-terminal and N-terminal fusion constructs between two orthogonal dCas9 enzymes (d*Sp*Cas9 and d*Sa*Cas9) and LgBiT and SmBiT of NanoLuc luciferase. N-terminal LgBit-d*Sp*Cas9 and SmBit-d*Sp*Cas9 fusion proteins contain HA epitopes; all other fusion proteins contain the 3× FLAG epitope. All constructs have two nuclear localization signals (NLS). (**B**) Heatmap representing SBR defined as normalized NanoLuc luminescence of on-target sgRNA pairs divided by the normalized NanoLuc luminescence of control “non-loop” sgRNA pairs. Eight fusion protein combinations of the dual dCas9 species DNA biosensor are listed in rows, and 24 different orientations and spacing combinations for *MUC4* sgRNA pairings are listed in columns. sgRNA pairs are spaced in tandem, inverted, or everted orientation. Heatmaps show apparent SBRs for each sgRNA pair (*n *= 4).

Previously, we demonstrated the ability to image unique non-repetitive DNA sequences in individual cells using a d*Sp*Cas9-based split NanoLuc luciferase biosensor [21]. Compared to “always-on” fluorescent reporters, the advantages of this system include low background in the absence of DNA binding and enzymatic amplification of the signal when both halves of the split system are bound next to each other. Here, we used an enhanced biosensor that used two orthogonal dCas9 proteins that recognize different PAM sequences to avoid unproductive binding orientations (Fig. 1A and Supplementary Fig. S1). Optimization of the detection system included several variables totaling 192 different iterations. All possible combinations of d*Sp*Cas9 (PAM 5′-NGG-3′) and d*Sa*Cas9 (PAM 5′-NNGRRT-3′) with a split luciferase component (LgBiT or SmBiT) appended to either the N- or C-terminus were assayed on DNA target sites juxtaposing the dCas9 proteins at eight different spacings (∼5–45 bp) and in three orientations (tandem, inverted, and everted) on the endogenous *MUC4* locus in HEK293T cells (Fig. 1A and Supplementary Fig. S1A). Plasmid DNA expressing the biosensor components was co-transfected along with d*Sa*Cas9 and d*Sp*Cas9 sgRNA pairs. A GFP reporter plasmid was additionally co-transfected to assess transfection efficiency. Fluorescence and luminescence were measured with a luminometer 24 h post-transfection, and SBR ratios were calculated relative to a control “non-loop” sgRNA pair that binds non-interacting regions in the genome. From 192 conditions tested, the optimal biosensor combination was found to be LgBiT-d*Sa*Cas9 + d*Sp*Cas9-SmBiT in tandem orientation with various spacings (Fig. 1B). The greatest SBRs (∼7–9-fold) were obtained at the highest concentration (10 fmol) of biosensor plasmid used in cell transfection (Supplementary Fig. S1B**)**.

Despite the substantially lower autoluminescent background compared to autofluorescent background in cells, the absolute signal from a live cell split luminescent reporter is several orders of magnitude dimmer compared to the absolute signal observed with full fluorescent reporters. In principle, a hybrid system with the high brightness of a fluorescent system and the low cellular background of the luminescent systems would be ideal. We therefore evaluated a different biosensor design called NanoBRET, in which the NanoLuc bioluminescence was used to excite a HaloTag618 fluorophore using bioluminescence resonance energy transfer (BRET). In the NanoBRET process, a low-background, glow-type luminescent signal is amplified to produce a bright fluorescent signal without the need for exogenous excitation light. Thus, we created eight directional fusion constructs (Supplementary Fig. S2A) between HaloTag and NanoLuc luciferase and d*Sp*Cas9 and d*Sa*Cas9. DNA constructs for this dual dCas9 species NanoBRET DNA biosensor were transiently co-transfected with the same sgRNA spacings and orientations targeting unique sites in the *MUC4* locus in HEK293T cells (Supplementary Fig. S2B). Luminescent and fluorescent signals were measured using a luminometer. Strikingly, we found that only the combination of HaloTag-d*Sp*Cas9 with d*Sa*Cas9-NanoLuc was efficient in eliciting both higher NanoBRET efficiency and NanoLuc luminescence, with the SBR reaching up to 4.8-fold relative to control “non-loop” sgRNA pairs. However, this approach was ultimately found too restrictive of spacing for our purposes. We therefore continued our study with the split NanoLuc biosensor.

### Biosensing of chromatin loops at the *MYC* TAD boundaries and at cell-type specific promoter-super enhancer loops

**Figure 2.**
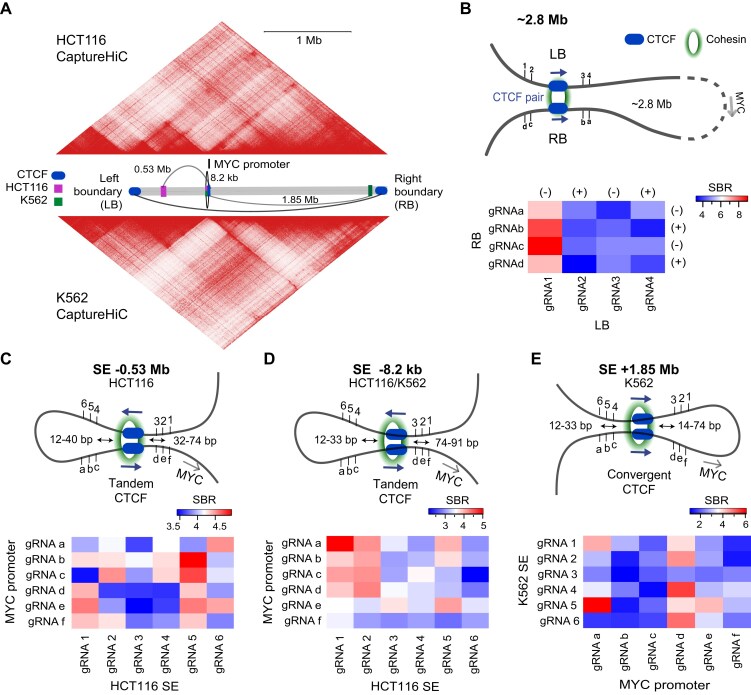
Biosensing of chromatin loops at the *MYC* TAD boundaries and at cell-type-specific promoter–super enhancer loops. (**A**) Region capture Micro-C maps of the *MYC* TAD locus in HCT116 and K562 cells. Cartoon in the center depicts the ∼2.8 Mb *MYC* TAD. *MYC* promoter–super enhancer loops targeted with the LgBiT-d*Sa*Cas9 + d*Sp*Cas9-SmBiT DNA biosensor are indicated in addition to *MYC* TAD left (LB) and right (RB) boundary regions. Magenta and green rectangles represent super enhancer regions in HCT116 and K562 cells, respectively. Blue ovals represent all CTCF binding sites outside super enhancer regions. (**B**) Representation of chromatin loop between left and right *MYC* TAD boundaries. sgRNAs targeting the left boundary are labeled 1–4, and sgRNAs a–d target the right TAD boundary. Heatmap represents chromatin loop biosensing results from 16 pairs of sgRNAs tiling along the pair of convergent CTCF binding sites. Heatmap shows SBR, which is defined as normalized NanoLuc luminescence of sgRNA pairs divided by the normalized NanoLuc luminescence of control “non-loop” sgRNA pairs. (C–E) Cartoon representations of intra-TAD chromatin loops between the *MYC* promoter and SE regions in HCT116 or K562 cells. Super enhancers 0.53 Mb (**C**) and 8.2 kb (**D**) upstream of the MYC promoter, as well as 1.85 Mb (**E**) downstream of the MYC promoter, were evaluated. sgRNAs were given labels a–f at the *MYC* promoter binding sites and labels 1–6 at the cell type-specific SE binding sites. Heatmaps summarize chromatin loop biosensing results from 36 pairs of sgRNAs tiling along a CTCF binding site within the large SE and tiling along a highly conserved CTCF binding site at the *MYC* promoter. Heatmaps show apparent SBRs for each sgRNA pair (*n *= 4). Individual SBR scales are shown at top right for each heatmap.

Based on the LgBiT-d*Sa*Cas9 + d*Sp*Cas9-SmBiT biosensor that produced a signal upon binding in close proximity on linear DNA, we hypothesized that chromatin loops could be detected if the biosensor components could be brought into close proximity by binding the DNA at the base of the chromatin loop. To model whether the distances between DNA strands at the base of chromatin loops could be feasible for this purpose, we first created an *in silico* cohesin-CTCF homodimer-anchored chromatin loop model at positions adjacent to homodimerized CTCF proteins (Supplementary Fig. S3) using a combination of known crystal and NMR structures and a full predicted structure from AlphaFold (see Supplementary Note 1). The model suggested a relatively short distance (∼0.7–4 nm) between *cis*-interacting DNA sequences (Supplementary Fig. S4), potentially facilitating reassembly of the split biosensor. For experimental validation, we chose the *MYC* TAD (Fig. 2A) because chromatin interactions between the *MYC* promoter region and various cell type-specific super enhancers (SEs) within this TAD have been thoroughly characterized across many common cancer cell lines [24, 30–36].

TAD boundaries are enriched in convergently oriented CTCF binding sites [37, 38], which often represent the strongest chromatin contact regions [20, 39, 40] and insulate super enhancer regions from surrounding TADs [41]. We first examined whether strong TAD boundary loops with convergently oriented CTCF proteins could be detected with the dual biosensor technology. For these experiments, we chose a pair of boundary CTCFs that anchor an insulated neighborhood (hg38 chr8:126 837755–129 737754) encompassing *MYC* and all interacting super enhancers (Fig. 2B). The CTCF pair is hence separated by ∼2.8 Mb.

We began by designing a set of four d*Sp*Cas9 and d*Sa*Cas9 sgRNAs surrounding one of the CTCF pairs. We combined pairings of sgRNAs with approximately equal spacing from the CTCF binding site as much as possible given PAM site frequency constraints in this region. In addition, we tested combinations of equally spaced sgRNAs on both strands and on either side of the loop anchor. Plasmid DNA expressing the biosensor components was co-transfected along with all possible combinations of d*Sa*Cas9 and d*Sp*Cas9 sgRNAs in addition to the GFP reporter plasmid. Fluorescence and luminescence were again assessed with a luminometer 24 h post-transfection. Background luminescence levels were measured using a control “non-loop” sgRNA pair that binds non-interacting genomic regions. We observed a range of SBR ranging from 3.3 to 9-fold when targeting the TAD boundary loop in K562 cells (Fig. 2B). The highest SBR of nine-fold was obtained when sgRNA 1 at the left boundary region (−strand, −153 bp from CTCF) was paired with sgRNA c at the right boundary region (−strand, +62 bp from CTCF). These results strongly suggested the ability to monitor chromatin conformations using the split luciferase system.

To further confirm the functionality of our DNA biosensor system, we tested a second CTCF pair. Rewardingly, similar results were obtained when targeting a second CTCF pair 5 kb from the initial boundary CTCF pair (Supplementary Fig. S5A and B). We designed four sgRNAs specific to the left boundary region and eight sgRNA pairs spanning the right boundary region. We tested 16 sgRNA pairings at the second boundary CTCF pair and observed SBRs ranging from 3.1- to 10.6-fold (Supplementary Fig. S5C). SBRs were comparable for innermost (<50 bp) and outermost (>100 bp) gRNA pairings. The highest SBR of 10.6-fold was measured when sgRNA 2 at the left boundary region (+strand, +49 bp from CTCF) was paired with sgRNA e at the right boundary region (+strand, +41 bp from CTCF). A similar result of 8.3-fold SBR was observed when sgRNA 3 at the left boundary region (+strand, +179 bp from CTCF) was paired with sgRNA g at the right boundary region (+strand, +166 bp from CTCF). Together these data support the hypothesis that our biosensor technology can detect individual chromatin interactions between TAD boundary elements.

We next wanted to investigate whether the dual-species biosensor can also detect chromatin loops between the *MYC* promoter and cell-type-specific SE that are located within the *MYC* TAD boundaries. We specifically picked three major interactions between a strongly conserved CTCF binding site upstream of the *MYC* promoter and distal SE regions in HCT116 and/or K562 cells (Fig. 2A), which had been shown to be strongly interacting regions in these cell lines from HiChIP and ChIA-PET data sets [24, 30, 31]. There is a tandem ∼0.53 Mb *MYC* promoter–super enhancer loop specific to HCT116 cells, a small tandem ∼8.2 kb *MYC* promoter–super enhancer loop that is common to both HCT116 and K562 cells, and a convergent ∼1.85 Mb *MYC* promoter–super enhancer loop specific to K562 cells (Fig. 2A). We confirmed cell-type-specific loops in HCT116 and K562 cells using 4C-seq with a non-blind viewpoint centered on the conserved CTCF binding site upstream of the *MYC* promoter (Supplementary Fig. S6A).

For each *MYC* promoter–super enhancer loop, plasmid DNA expressing the biosensor components was co-transfected along with all possible combinations of six d*Sa*Cas9 sgRNAs and six d*Sp*Cas9 sgRNAs that targeted the *cis*-interacting DNA backbones at loop anchors. A GFP reporter plasmid was used again to compute transfection efficiency. To establish background luminescence levels, a control “non-loop” sgRNA pair was co-transfected to bind noninteracting regions of the genome. We observed SBRs ranging from 3.3- to 5.0-fold when the DNA biosensor was directed to bind the ∼0.53 Mb *MYC* promoter–super enhancer chromatin loop in HCT116 cells (Fig. 2C). The highest signal resulted from pairing sgRNA 5 at the super enhancer with sgRNA b at the *MYC* promoter. We next assayed the much smaller ∼8.2 kb loop upstream of the *MYC* promoter in HCT116 cells. We observed a range of SBRs between 2.1- and 5.0-fold (Fig. 2D). Since this closer super enhancer is present in both HCT116 and K562 cells, we applied a set of 12 sgRNA pairs that produced the highest SBRs in HCT116 cells to the same *cis* interaction in K562 cells. Similar to HCT116 cells, we observed an SBR range of 1.7- to 5.9-fold (Supplementary Fig. S7). In both cell lines, SBR was highest when enhancer sgRNA 1 was paired with promoter sgRNA a at 5.0-fold in HCT116 cells and 5.9-fold in K562 cells. Finally, when our biosensor was directed to the distant ∼1.85 Mb *MYC* promoter–super enhancer chromatin loop in K562 cells, we observed a range of SBRs of 1.1-fold to 6.1-fold (Fig. 2E). Pairing sgRNA 5 at the super enhancer with sgRNA a at the *MYC* promoter in K562 cells resulted in the highest SBR.

We also investigated whether the biosensor signal (SBR) correlates with 3D interactions observed by Hi-C methods. We took advantage of available high-resolution region capture Micro-C (RCMC) data for the *MYC* TAD locus. We calculated VC-normalized contact scores for each gRNA pair using 50 bp and 200 bp bins overlapping gRNA coordinates. Due to resolution limitations, smaller 50 bp bins had few overlaps and were not suited for analysis. We therefore used 200 bp bins to compare Micro-C contact frequencies with SBR signal for each gRNA pair from our biosensing experiments (Supplementary Fig. S8 and  Supplementary Table S1). We did not observe any correlation.

In summary, our split dual-biosensor effectively detects chromatin loops, and our results show its versatility both at TAD boundaries and between proximal and distal regulatory elements.

### Live-cell imaging of *MYC* promoter–super enhancer loops

**Figure 3.**
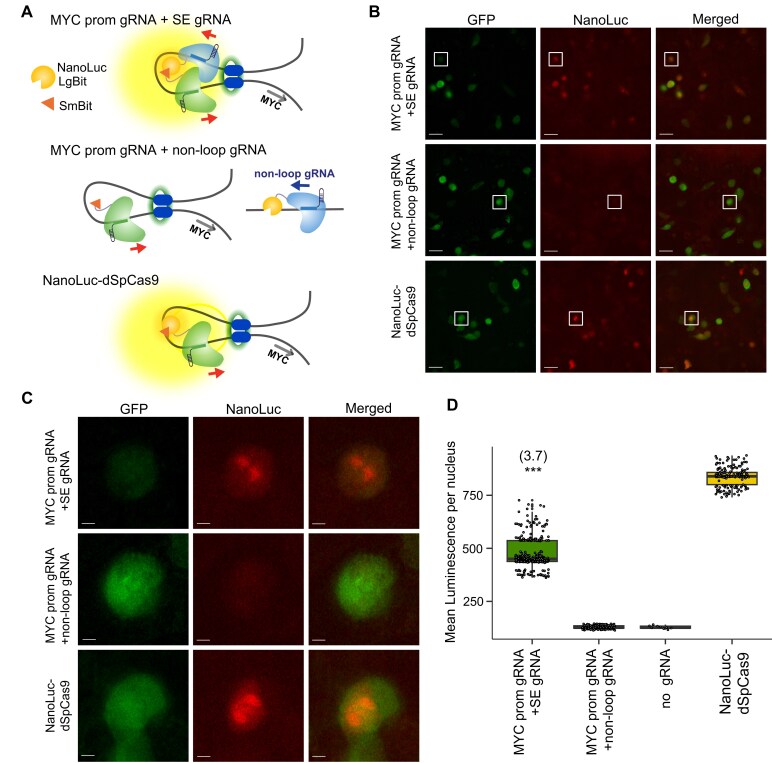
Imaging *MYC* promoter-super enhancer loops in live cells using low-light luminescence microscopy. (**A**) Cartoon depicting biosensor targeting the intra-TAD loop between the *MYC* promoter region and the super enhancer region ∼0.53 Mb upstream from the *MYC* promoter in HCT116 cells. SE sgRNA 5 paired with *MYC* promoter sgRNA b is compared to a control “non-loop” sgRNA pair and a NanoLuc-d*Sp*Cas9 control. Although a single allele is illustrated, luminescence signal is measured per nucleus, not per individual allele. (**B**) Representative live cell microscopy images of split biosensor at chromatin loop between *MYC* promoter and −0.53Mb SE. GFP fluorescence (green), NanoLuc luminescence (red), and merged images were taken on the Andor Dragonfly 200 Multi-modal Confocal System at 63X magnification. Signal from the *MYC* promoter and SE sgRNA pair is compared to a control “non-loop” sgRNA pair and a NanoLuc-d*Sp*Cas9 positive control. Scale bars = 25 μM. (**C**) 25 μM x 25 μM white panels from individual GFP fluorescence (green), NanoLuc luminescence (red), and merged images in panel (**B**) magnified. Scale bars = 3 μM. (**D**) Signal quantification per nucleus (36 h post-transfection) for the same *MYC* promoter and SE sgRNA pairs using the dual dCas9 species DNA biosensor. Apparent signal-to-background ratio for the ∼0.53 Mb super enhancer sgRNA 5 + *MYC* promoter b sgRNA pair is listed in parentheses. Unique nuclei were quantified for four conditions: (i) *MYC* promoter-SE sgRNA pair, (ii) *MYC* promoter plus non-loop targeting sgRNA, (iii) no sgRNA, and (iv) NanoLuc-d*Sp*Cas9 (*n *= 325, *n *= 143, *n *= 9, and *n *= 193 unique nuclei, respectively). Whisker plots show the median and interquartile range (IQR). Comparisons between group medians were made using an unpaired two-sided Student’s *t*-test (*P < .05; **P < .01; ***P < .001).

While the luminometer was useful to rapidly assess sgRNA pairs, the measurement represents the average of the bulk sample. To assess nuclear luminescence signals in individual live cells, we used the Andor Dragonfly 200 Multi-modal Confocal System to gather high-resolution images of the biosensor and top-ranked sgRNAs 5 and b targeting the ∼0.53 Mb loop in HCT116 cells (Fig. 2C and 3A). Negative controls included the control “non-loop” sgRNA pair, as well as no sgRNAs. A positive control was an equimolar amount of a NanoLuc-d*Sp*Cas9 fusion protein with sgRNAs to a known *cis*-interacting region to indicate maximum signal (Fig. 3A). The nuclei of many cells transfected with our biosensor showed bright luminescent areas, while cells transfected with noninteracting sgRNA pairs showed no luminescent areas and a diffuse signal in the nucleus (Fig. 3B and C). Analysis of individual nuclear signals showed a 3.7-fold SBR compared to our non-interacting background condition (Fig. 3D). In addition, comparison with the positive control revealed ∼57% of maximum theoretical signal output for our biosensor.

Our biosensor technology enables the direct visualization of chromatin loop signals in real time in individual live cells. This capability enables the examination of the presence or absence of specific chromatin loops in single cells and thereby has the potential to provide valuable insights into their dynamics. However, for proof-of-principle, all further experiments were performed in bulk live cells using the NanoLuc bioluminescence assay.

### Real-time monitoring of chromatin loop dynamics

The utility of a chromatin loop detection system depends on, at a minimum, the ability to discriminate between the presence or absence of a loop. To demonstrate this, we utilized an isogenic cell line with an enhancer deletion. Specifically, we investigated a well-characterized chromatin loop between the *MYC* promoter and the E7 enhancer region ∼0.34 Mb upstream of the *MYC* promoter in HCT116 cells [42]. As a negative control, we used an edited HCT116 cell line with the E7 enhancer region deleted [43]. Using the dual biosensor and the NanoLuc bioluminescence assay in wild-type HCT116 cells, we observed SBRs ranging from 1.6- to 3.4-fold (Supplementary Fig. S6B). As expected, when the biosensor was targeted to the same loop anchor regions in the E7 enhancer deletion HCT116 cells, we observed near background signal (0.99- to 1.16-fold SBR).

**Figure 4.**
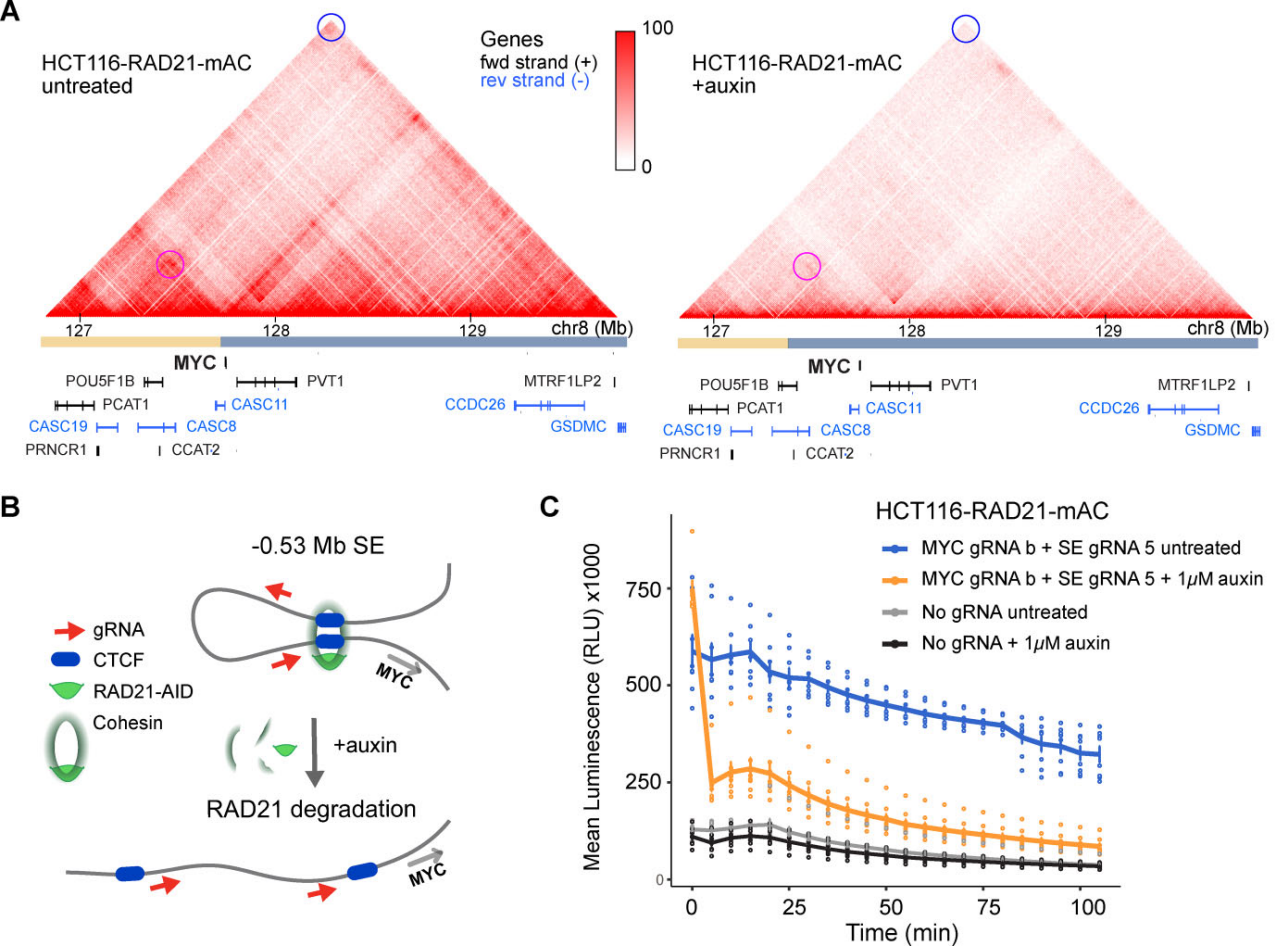
Real-time monitoring of chromatin loop dynamics using an AID system for endogenous RAD21 in HCT116 cells. (**A**) Hi-C contact map at 10 kb resolution showing the *MYC* TAD in untreated HCT116-RAD21-mAC cells (left) and after 6 h of auxin treatment (right), demonstrating general loss of chromatin loops. The TAD boundary and super enhancer interactions are highlighted with blue and magenta circles, respectively. The intensity of each pixel represents the normalized number of contacts between a pair of loci. Maximum intensity is indicated in the scale at the right. (**B**) Cartoon depicting loss of chromatin loop after inducing RAD21 degradation. (**C**) HCT116-RAD21-mAC cells treated with 1 µM auxin (5-Ph-IAA) for 105 min were compared to untreated HCT116-RAD21-mAC cells over the same 105 min time course. Both treated and untreated conditions were transfected with plasmids expressing LgBiT-d*Sa*Cas9 + d*Sp*Cas9-SmBiT and a *MYC* promoter–super enhancer gRNA pair (gRNA b and gRNA 5, respectively) targeting the −0.53 Mb super enhancer. Untreated and auxin-treated conditions, where no sgRNA pairs were transfected, are shown for comparison. Luminescence was measured (*n* = 8) in bulk live cells every 5 min for 105 min for each transfection condition.

Having demonstrated the ability to detect the presence or absence of a distinct chromatin loop, we next investigated whether the biosensor could report on chromatin dynamics over time, such as the loss of a loop. For these experiments, we assessed the same ∼0.53 Mb *MYC* promoter–super enhancer loop examined previously (Fig. 2C), but in an HCT116 cell line, HCT116-RAD21-mAC OsTIR1(F74G), that contained an inducible RAD21 degradation system. We refer to this line as HCT116-RAD21-mAC. The degradation system consisted of a second-generation auxin-inducible degron 2 (AID2) [44], an improved version of the originally described auxin-inducible degron (AID) technology [45]. It was previously demonstrated that loss of cohesin subunit RAD21 eliminated all loop domains immediately after auxin treatment of HCT116 cells harboring an RAD21-AID endogenous system (Fig. 4A-B) [46]. In addition, using 4C-seq with a non-blind viewpoint centered on the conserved CTCF binding site upstream of the *MYC* promoter, we confirmed that loop domains originally present in HCT116-RAD21-mAC cells were reduced to near background levels after 120 min auxin treatment using the 5-Ph-IAA ligand (Supplementary Fig. S9A). Addition of ligand in HCT116-RAD21 cells leads to degradation of RAD21 and dissolution of 3D architecture [46]. To evaluate dynamics of chromatin loop degradation, luminescence was monitored over time with or without the addition of 5-Ph-IAA ligand to the cell culture medium. We monitored luminescence every 5 min in real time in HCT116-RAD21-mAC cells targeting the −0.53 Mb SE upstream of the MYC promoter CTCF binding site (see also Figs 2C and 3). Using this AID2 degron system for RAD21, we observed that the luminescent signal rapidly decreased to near background levels when HCT116-RAD21-mAC cells were treated with 1 µM 5-Ph-IAA for 105 min compared to untreated HCT116-RAD21-mAC cells over the same time course (Fig. 4C). Approximately 67% of the initial luminescent signal at t0 was degraded in the first 5 min upon treatment with 1 µM 5-Ph-IAA, while ~4% of the initial luminescent signal at t0 was degraded in untreated cells. The rapid decrease in luminescent signal after treatment, reflecting chromatin loop degradation, is consistent with the extremely short half-life of ∼12 min for RAD21-mAID-Clover reported upon treatment of HCT116-RAD21-mAC cells with 1 µM 5-Ph-IAA [44]. Untreated HCT116-RAD21-mAC cells showed natural decay of the bioluminescence signal over time, which was comparable to experimental controls using wild-type HCT116 cells and two different MYC promoter-super enhancer gRNA pairs (Supplementary Fig. S9B). Decay of luminescence with time was observed in both untreated and auxin-treated HCT116 cells (Supplementary Fig. S9C).

In summary, we demonstrated that the dual biosensor can detect chromatin loop presence or absence in live cells, and more importantly monitor chromatin dynamics in real-time. This capability allows for a deeper understanding of chromatin structure and function, enabling us and other researchers to track changes in chromatin loops as they occur.

## Discussion

In this study, we developed a dual dCas9 species DNA biosensor with real-time monitoring ability of the 3D chromatin landscape. In particular, we hypothesized that our dual biosensor could provide a reasonable approach for the detection of chromatin loops if the expected distance to bridge the *cis*-interacting genomic regions at the loop anchor was short enough to allow reassembly of NanoLuc luciferase. Our results demonstrate that our biosensor can detect the presence of chromatin loops in living cells at high sensitivity, up to 10.6-fold above background levels. In addition to detecting the *MYC* TAD loop boundaries (∼2.8 Mb), we demonstrate biosensing capability for chromatin loops between the *MYC* promoter and four cell-type-specific (super) enhancers at different distances (ranging from 8.2 kb to 1.85 Mb). Thus, we believe that our models that predict a relatively short ∼0.7–4 nm distance between *cis*-interacting DNA sequences at the base of a loop anchor immediately adjacent to homodimerized CTCFs are accurate, making them highly amenable to targeting by DNA biosensors in live cells in real time. The design features could be useful for the creation of similar split-reporter biosensors, such as with split fluorophores [47–49].

Ideally, real-time biosensing should not interfere with loop structure. Therefore, the positioning of biosensor binding in relation to CTCF-occupied sites needs to be considered. The highest SBRs were generally observed for sgRNA pairings 100–150 bp from the CTCF homodimer. A notable exception was the pairing of sgRNA 4 at the +1.85 Mb super enhancer and sgRNA d at the *MYC* promoter in K562 cells, a pairing immediately adjacent to the CTCF homodimer. This sgRNA pairing produced the second highest SBR of 5.5-fold at this chromatin loop in K562 cells. Notably, the sgRNAs for this pair were separated from the nearest CTCF site by 33 and 74 bp, respectively, which likely provided enough space to minimize steric hindrance effects with CTCF that could negatively affect the efficiency of biosensor binding. Taken as a whole, the data suggest that a spacing of at least 30–40 bp between CTCF and the nearest sgRNA is necessary to achieve sufficient signal-to-background in live cells using our DNA biosensor. However, signal-to-background might be improved by increasing the distance to at least 200–225 bp. Future testing should determine whether there is a maximum allowable distance threshold for targeting cohesin and CTCF homodimer-anchored chromatin loops.

Due to the nature of the biosensor technology and Hi-C, we cannot directly compare our SBR values to contact frequencies. A major limitation is Hi-C resolution, even when we are using region capture Micro-C data at 50-bp bins. For example, only 1 out of the 16 gRNA pairs spanning the left and right boundary elements of the MYC TAD (Fig. 2B) produced a detectable contact score. Most gRNAs target sites within 200 bp or less of the interaction site, and thus 200-bp bins have the caveat that several gRNAs fall within the same bin. In addition to contact frequency, SBR signal is also influenced by factors such as gRNA efficiency, proximity to the interaction site, and potential interference with other chromatin features. While these factors may influence signal strength, nearly all gRNA pairs were successful in generating NanoLuc signal. We observed the strongest SBR signals at TAD boundaries. However, Hi-C and Micro-C data typically show the opposite trend, as interaction frequencies decrease with increasing genomic distance.

Most importantly, we were able to monitor real-time chromatin loop dynamics in live cells using a cell model with inducible RAD21 degradation. In experiments targeting the critical RAD21 subcomponent of the cohesin complex for degradation using a novel auxin ligand (5-Ph-IAA), we found that when RAD21 was degraded, the resulting signal outputted by our chromatin loop biosensor dropped rapidly within 5–10 min. This is in the same general range as the 20–40-min loop loss observed by Rao *et al.* [46] upon auxin-induced cohesin degradation and 10–30-min loop lifetime observed by Gabriele *et al.* [7] using multiple knocked-in fluorescently tagged loop flank sequences in live cells. Thus, RAD21 degradation and cohesin complex dissolution were sufficient to decrease the signal produced by our biosensor at the ∼0.53 Mb super enhancer–MYC promoter loop. Indeed, our independent 4C-seq analysis using the MYC promoter CTCF binding site as a viewpoint showed that raw 4C-seq signal was reduced to near-background levels across the ∼2.8 Mb MYC TAD, and notable 4C-seq peaks were ablated as a result of 5-Ph-IAA treatment. We demonstrate that our biosensor technology can dynamically monitor 3D chromatin interactions with the flexibility to be reprogrammed to target any chromatin interaction of interest simply by modifying the guide RNA pairs.

We detected chromatin loops in live cells using two complementary approaches: luciferase-based assays (plate reader) and microscopy. NanoLuc luminescence assays measure chromatin loop signals in bulk cell populations. At individual time points, these signals recapitulate loops identified by Hi-C or 4C, but the biosensor system allows for simpler, higher-throughput evaluation of individual interactions. Importantly, the split biosensor system is non-destructive, enabling monitoring of cell populations over time or in response to treatment. Using microscopy, we are able to detect specific chromatin loops in single live cells. Our experiments have not employed allele-specific gRNAs, and therefore the luminescence signal in the nucleus does not resolve allele-specific chromatin loops. This could, however, be achieved by designing gRNA pairs targeting SNPs, insertions, or deletions at loop interaction sites where possible.

In conclusion, this study describes a powerful new tool for studying chromatin dynamics and their role in gene regulation and other cellular processes. Our “turn-on” split luciferase biosensor system has advantages over “always-on” fluorophore-based systems that have a high background signal due to the presence of unbound fluorophores or random fluorescent protein accumulation [50]. To achieve sufficient signal over background, such methods typically require 26–36 closely spaced sgRNAs [51–53] or complex microscopy techniques and specialized equipment. Other fluorescently labeled studies focused on TAD dynamics used genome editing to homozygously label the CTCF sites with specific sequence arrays, which were then visualized by co-expressing fluorescently tagged binding proteins [7]. In contrast, we typically achieved SBRs of 3–10-fold using a single pair of gRNAs in unmodified live cells using instrumentation as simple as a luminometer. The split luciferase biosensing platform should therefore find broad use for live-cell chromatin studies.

## Supplementary Material

gkaf1324_Supplemental_Files

## Data Availability

Plasmids expressing biosensor components using the split NanoLuc luciferase and NanoBRET systems are available on Addgene (LgBiT-d*Sa*Cas9 #236758, d*Sp*Cas9-SmBiT #236759, HaloTag-d*Sp*Cas9 #238946, dSaCas9-NLuc #238947). Sequencing data for 4C-seq have been deposited in the Gene Expression Omnibus (GEO) under accession # GSE295357.
