# Supplementary material for: A split luciferase biosensing platform for detection and imaging of chromatin loops in individual live cells
Source: Nucleic Acids Res. 2025 Dec 3;53(22):gkaf1324. doi: 10.1093/nar/gkaf1324 (PMC12673846; doi:10.1093/nar/gkaf1324)
Supplement: gkaf1324_Supplemental_Files [file gkaf1324_supplemental_files.zip › Supplementary Figures rev.pdf]

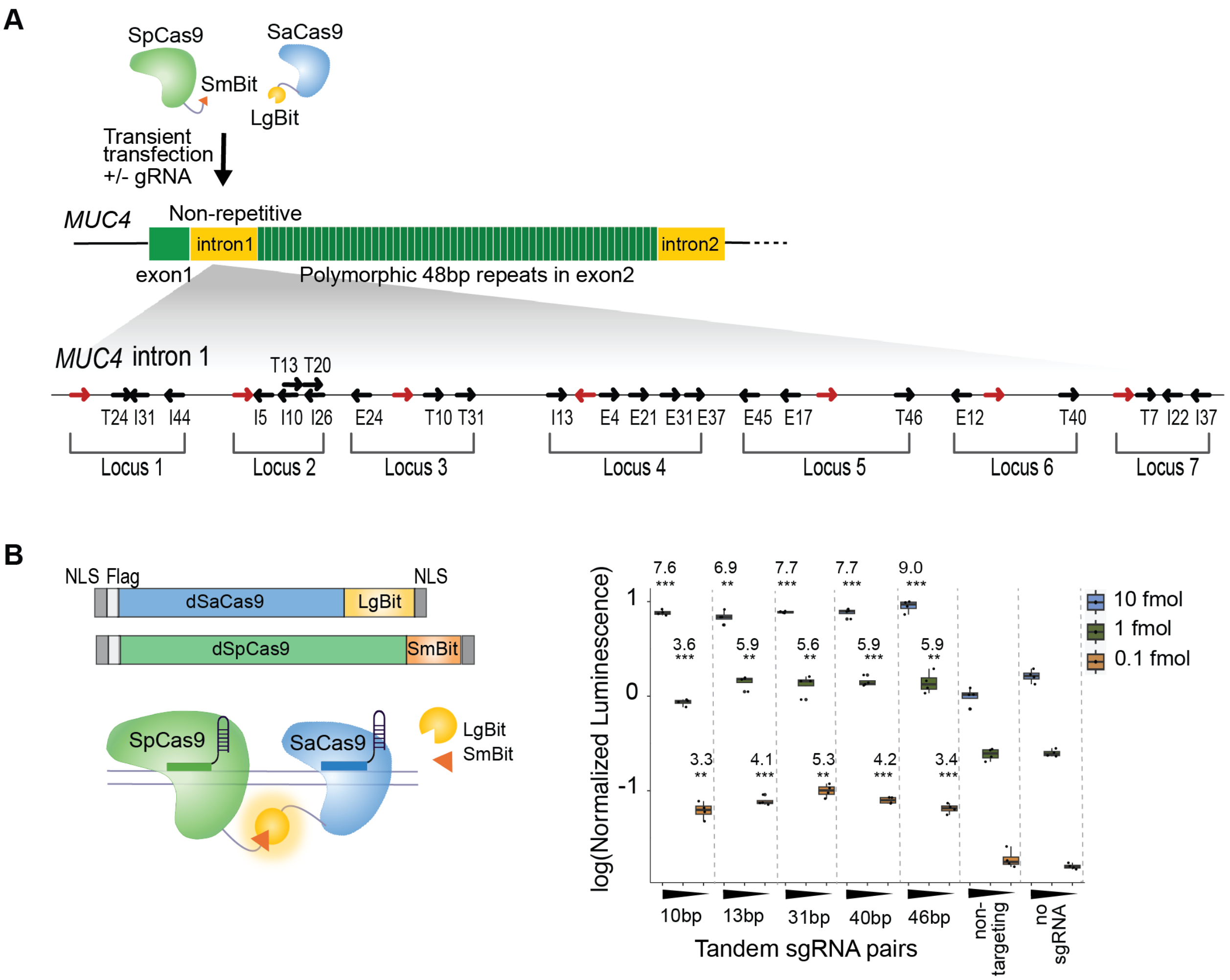

Supplementary Figure S1. Evaluation of split NanoLuc biosensor at non-repetitive *MUC4*. A. Cartoon of experimental setup for testing the dual dCas9 species DNA biosensor at the non-repetitive intron 1 of human *MUC4*. Binding locations and orientations for sgRNAs directed to bind seven loci within the non-repetitive *MUC4* intron 1 region. Orientations are given for d*Sp*Cas9 sgRNAs (black) relative to the d*Sa*Cas9 sgRNA (red) within each locus. Each cluster of sgRNA binding sites was separated from other clusters by between ~100 and 700 bp genomic distance. **B.** Cartoon depicting the LgBiT-d*Sa*Cas9 + d*Sp*Cas9-SmBiT DNA biosensor. Plasmids expressing biosensor components at 3 different concentrations are co-transfected with indicated sgRNA pairs. After 24 h luciferase activity was evaluated and shown as log-transformation of normalized luminescence signals were measured for the dual species LgBiT-d*Sa*Cas9 and d*Sp*Cas9-SmBiT DNA biosensor binding to several combinations of loci in the non-repetitive region of *MUC4* intron 1 in HEK293T cells at 10 fmol, 1 fmol, and 0.1 fmol probe transfected. Apparent signal-to-background ratios are shown relative to the non-targeting sgRNA pair. Data are presented as the mean ± s.e.m., $n = 4$. Significance was determined by unpaired two-sided Student's t-test (*$p < 0.05$; **$p < 0.01$; ***$p < 0.001$).

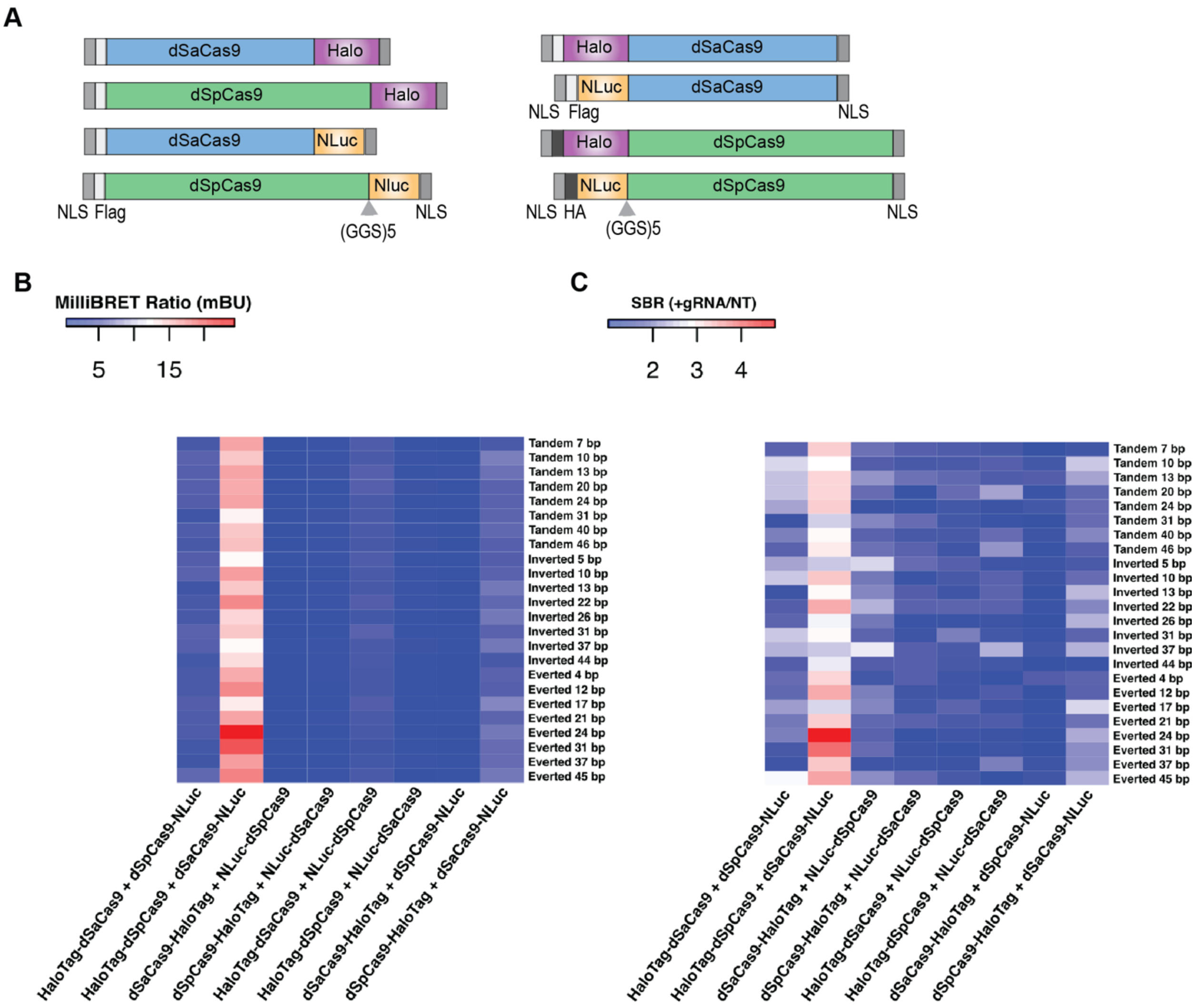

Supplementary Figure S2. **Evaluation of NanoBRET DNA biosensor at non-repetitive *MUC4*. A.** Cartoon representation of the C-terminal (left) and N-terminal (right) fusion constructs between two orthogonal dCas9 enzymes and HaloTag (Halo) and NanoLuc luciferase (NLuc). N-terminal NanoLuc-d*Sp*Cas9 and HaloTag-d*Sp*Cas9 fusion proteins contain HA epitopes, all other fusion proteins contain the 3X FLAG epitope. All constructs have two nuclear localization signals (NLS). **B.** Heatmap representing the NanoBRET efficiency given by the milliBRET ratio (mBU) defined as the HaloTag 618 ligand fluorophore emission value (RFU, 618 nm) divided by the NanoLuc luciferase donor luminescence (RLU, 460 nm) multiplied by 1000. **C.** Heatmap representing signal-to-background ratio (SBR) defined as normalized milliBRET ratio in NanoBRET biosensing conditions with sgRNA pairs transfected divided by the normalized milliBRET ratio in background conditions using control “non-loop” sgRNA pairs. Columns in heatmaps show fusion protein orientations of the NanoBRET DNA biosensor and rows show the 24 different orientation and spacing combinations for *MUC4* sgRNA pairs.

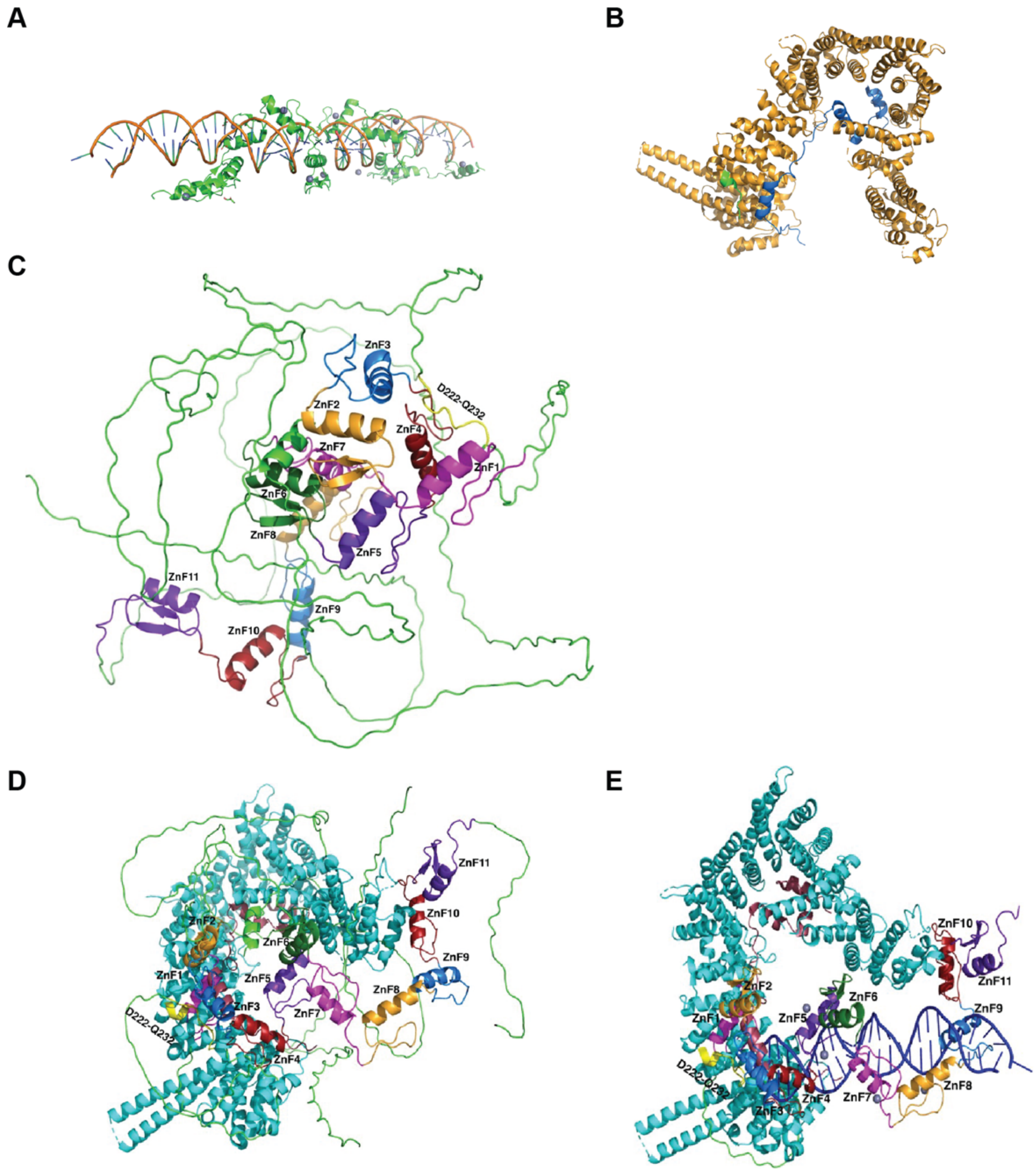

Supplementary Figure S3. Modeling the structure of cohesin and CTCF homodimerization-mediated loop anchors. A. Crystal structure of ZnFs 4-10 of CTCF (green) bound to 28-mer DNA (orange). **B.** Crystal structure of cohesin subunits SA2 (orange) and SCC1/RAD21 (blue) in association with the N-terminal loop domain of CTCF. CTCF amino acids D222-Q232 are shown in green, which interact with S334, I337, R338, and L341 of SCC1 and Y297, R298, and W334 of SA2. **C.** Full CTCF structure predicted by AlphaFold. CTCF amino acids D222-Q232 are highlighted in yellow within the N-terminal loop domain. ZnFs 1-11 in CTCF are labeled. **D.** Alignment of the structures which both contain the stretch of amino acids from positions 222-232 in the N-terminus of CTCF (yellow) to produce a full CTCF-SCC1-SA2 complex. Cohesin subunits SA2 (teal) and SCC1/RAD21 (maroon) are shown attached to CTCF via intermolecular interactions between specific amino acids in SCC1/RAD21 and CTCF. **E.** Alignment of the CTCF-SCC1-SA2 complex with ZnFs 4-10 with 28-mer CTCF binding site. Redundant ZnFs 4-10 and CTCF loop domains predicted by AlphaFold were removed after alignment. 28-mer DNA is shown in dark blue.

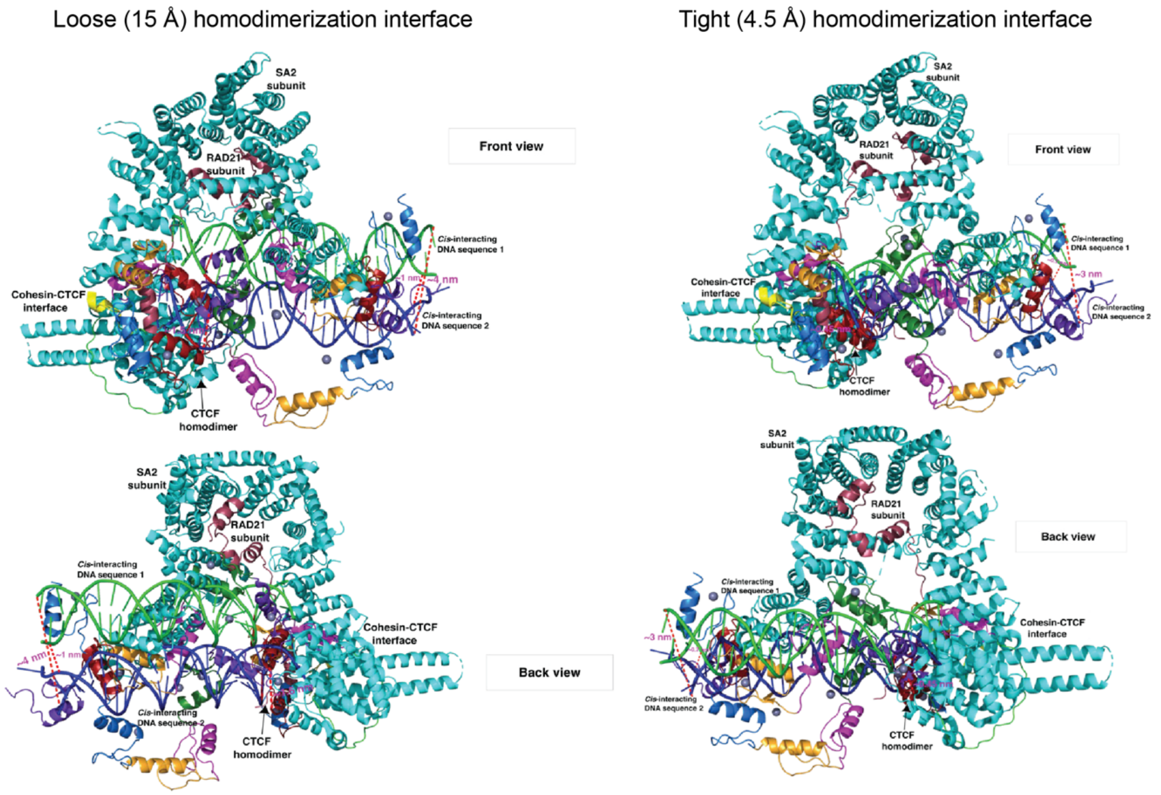

Supplementary Figure S4. Cohesin and CTCF-mediated loop anchor models for loose (15 Å) and tight (4.5 Å) homodimerization interface. Composite loop anchor model containing cohesin subunits SA2 (teal) and SCC1/RAD21 (maroon), homodimerized CTCF proteins with N- and C-terminal loop domains removed (multicolored) showing a 15 Å or 4.5 Å dimerization interface, and two *cis*-interacting DNA sites (dark blue and green). This structure is rotated 180 degrees around the vertical axis to depict a view from the back of the loop anchor (bottom). For both models, the cohesin-CTCF interface is highlighted in yellow and the CTCF homodimer interface at ZnF 4 (red) is highlighted. Distances between *cis*-interacting DNA sites are shown between positions ~11 bp upstream of the core 19 bp consensus CTCF binding site (upstream of sequence associated with ZnF 9) on the most distant and the most proximal pairings of strand positions, suggesting a specific range of expected distances between sgRNA binding sites for a DNA biosensor repurposed for chromatin loops of ~1-4 nm (loose) and ~0.7-3 nm (tight).

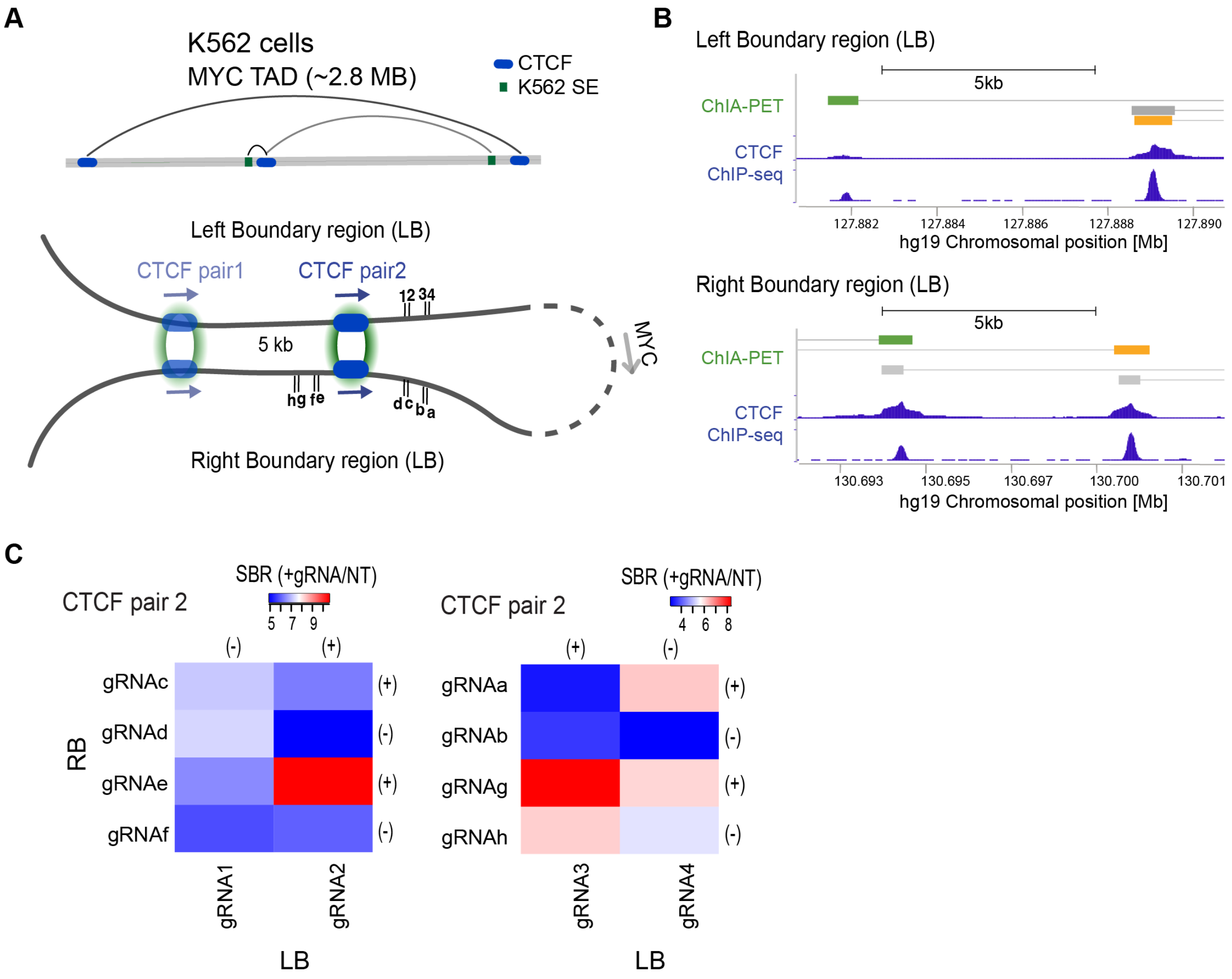

Supplementary Figure S5. Targeting boundary region chromatin loops at the *MYC* TAD using a dual dCas9 species LgBiT-d*Sa*Cas9 + d*Sp*Cas9-SmBiT DNA biosensor. A. Cartoon depicting the ~2.8 Mb *MYC* TAD boundary region in K562 cells. sgRNAs targeting CTCF pair 2 were given labels 1-4 and a-h at the *MYC* TAD left and right boundary region binding sites, respectively. **B.** UCSC genome browser track set showing K562 CTCF ChIA-PET and CTCF ChIP-seq signal tracks (aligned read density) from the ENCODE project at the *MYC* TAD left and right boundary region (LB, RB). **C.** Heatmaps representing chromatin loop biosensing results from 8 pairs of sgRNAs tiling along the downstream pair of convergent CTCF binding sites (CTCF pair 2). Luminescence and fluorescence were measured 24 h post-transfection. The innermost sgRNAs are between 27 bp and 49 bp (left heatmap) and the outermost sgRNAs are between 156 bp and 192 bp (right heatmap) from the CTCF binding site. sgRNA strand polarity is shown at top and right. Apparent signal-to-background ratios (comparisons made to non-targeting (NT) background condition where sgRNAs targeting non-interacting genomic regions selected based on public Hi-C data sets were transfected) are shown for each sgRNA pair, $n = 4$.

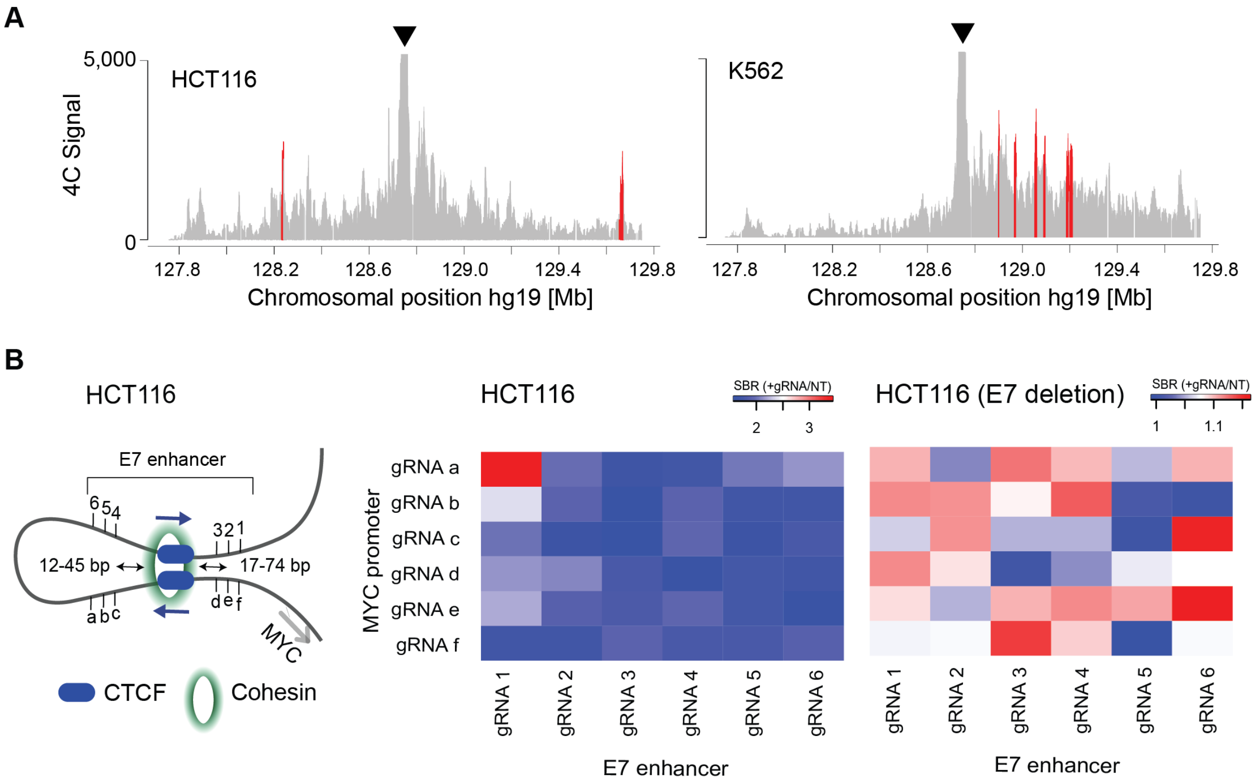

Supplementary Figure S6: Biosensing presence and absence of chromatin loops. A. 4C-seq validation of cell-type specific promoter-super enhancer loops. 4C signal is plotted by genomic position (hg19) for combined 4C-seq profiles of triplicate experiments with anchor at the conserved *MYC* promoter CTCF binding site (hg19 chr8:128,746,370, indicated as black triangle). Preferential chromatin loops ("peaks") are identified using the peakC R package and are shown in red for HCT116 cells (left) and K562 cells (right). **B.** Cartoon representation of intra-TAD chromatin loops between the *MYC* promoter and the E7 enhancer region in HCT116 cells. sgRNAs used for biosensing experiments are shown for each of the loop anchor regions (not drawn to scale). sgRNAs were given labels a-f at the *MYC* promoter binding sites and labels 1-6 at the E7 enhancer binding sites. Heatmaps show chromatin loop biosensing results in wild-type HCT116 cells (left) and the HCT116 E7 enhancer deletion cell line (right). 36 sgRNA pairs were targeted to the ~0.33 Mb *MYC* promoter-E7 enhancer chromatin loop and luminescence and fluorescence were measured 24 h post-transfection. Apparent signal-to-background ratios (SBRs) were determined relative to the background condition where a non-loop sgRNA pair targets non-interacting genomic regions. SBRs are shown for each sgRNA pair, $n = 4$.

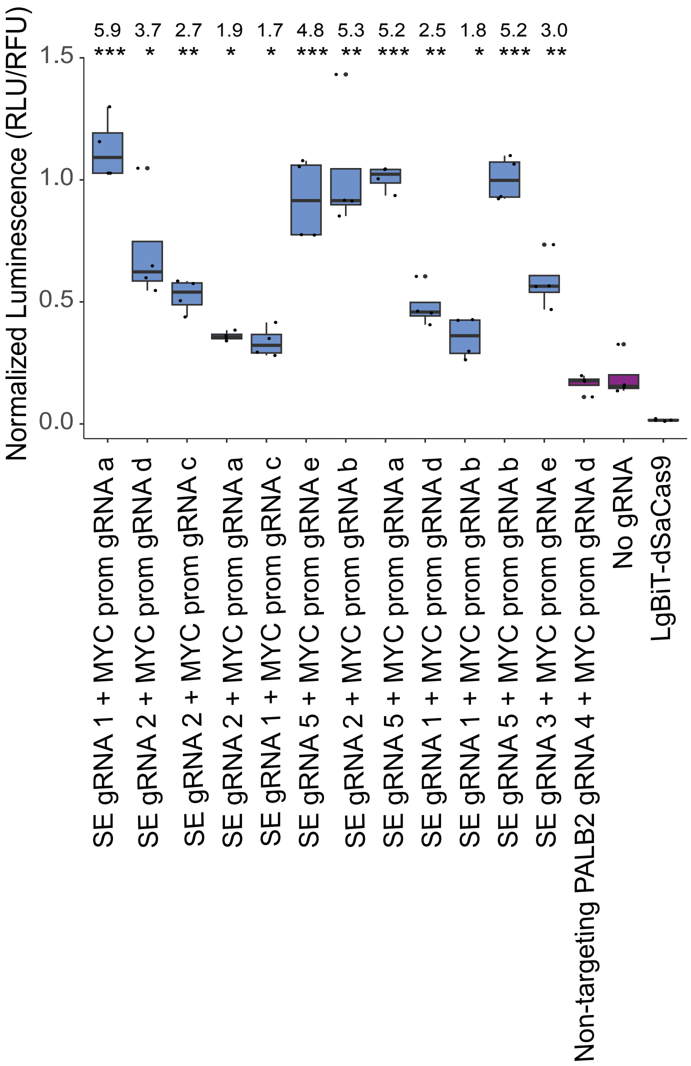

Supplementary Figure S7: Targeting promoter-super enhancer chromatin loops at the MYC locus using a dual dCas9 species LgBiT-dSaCas9 + dSpCas9-SmBiT DNA biosensor. Biosensing results in K562 cells targeting the ~8.2 kb MYC promoter-super enhancer chromatin loop. After co-transfection of plasmids expressing split biosensor components and 12 sgRNA pairs luminescence and fluorescence were measured 24 h later. Apparent signal-to-background ratios are listed above each sgRNA pair. A non-interacting sgRNA pair between the MYC promoter and *PALB2* were used as the background control. Data are presented as the median and interquartile range (IQR) and whiskers show dispersion from the IQR that is equal to the lesser of the 1st or 3rd quartiles plus or minus 1.5xIQR or the distance from the 1st or 3rd quartiles to the minimum or maximum points. Significance was calculated using unpaired two-sided Student's t-test (*$p < 0.05$; **$p < 0.01$; ***$p < 0.001$).

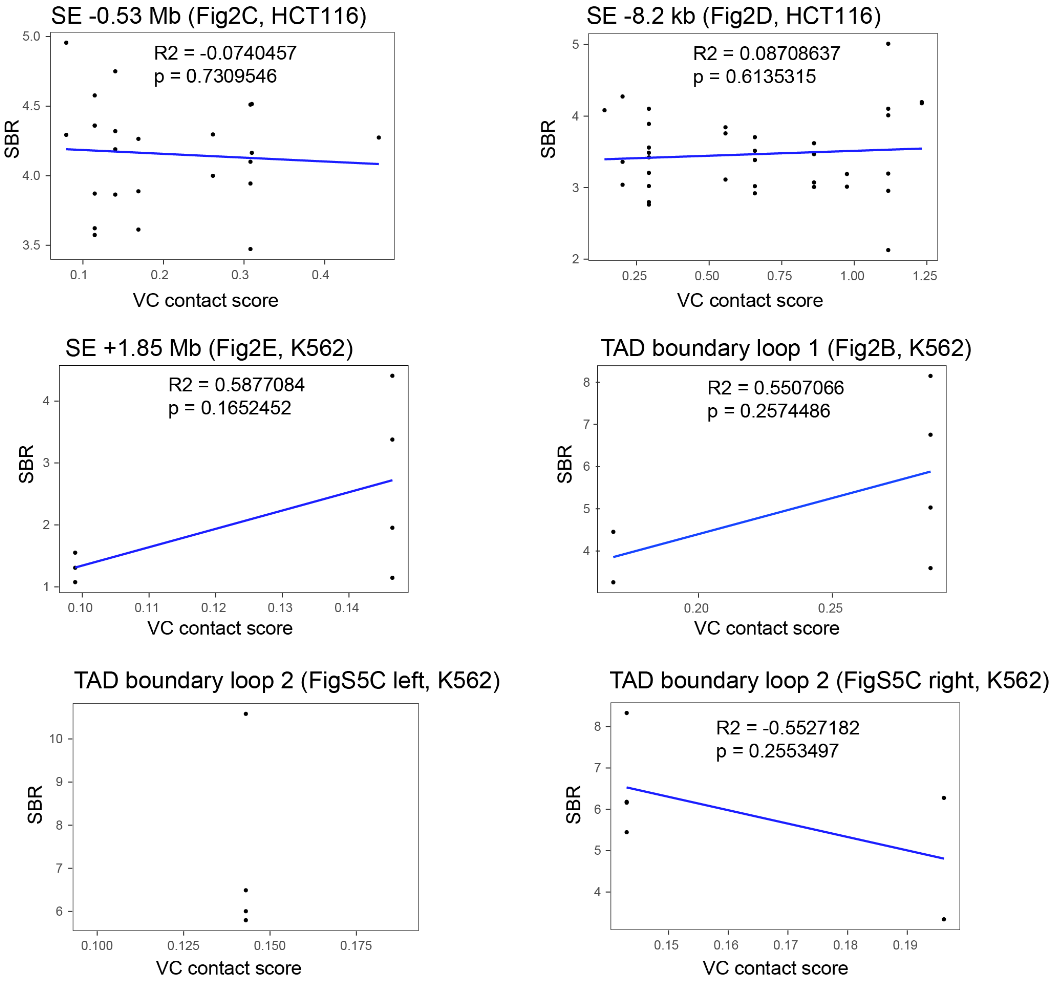

Supplementary Figure S8. Comparison between biosensor SBR and capture Micro-C contact scores. Normalized capture Micro-C contact scores (VC) were obtained for 200bp bins overlapping each gRNA of a gRNA pair. Each plot highlights results from chromatin loops between a *MYC* super enhancer (SE) and promoter or between the left and right *MYC* TAD boundaries. Signal-to-background ratios (SBR) from split biosensor assays (y-axis) are plotted against VC contact scores for each gRNA pair (x-axis), with R^2 coefficient and p-values indicated. Genomic coordinates for each gRNA pair, SBRs and VC scores are listed in Table S1. No correlation was observed between biosensor SBRs and capture Micro-C VC scores in HCT116 or K562 cells.

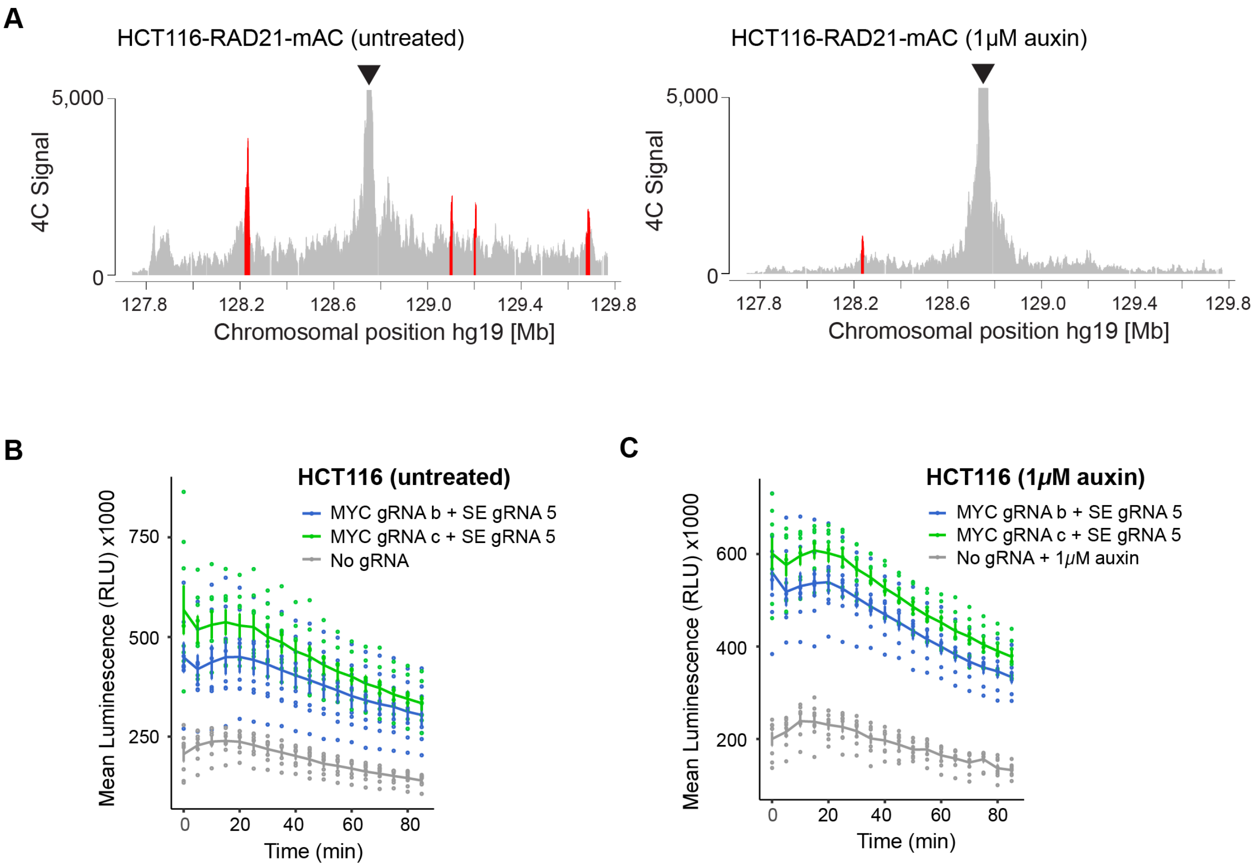

Supplementary Figure S9: 4C-seq validation of loss of promoter-super enhancer loops in auxin-inducible degron (AID) system for endogenous RAD21 in HCT116 cells (HCT116-RAD21-mAC). A. 4C signal is plotted by genomic position (hg19) for combined 4C-seq profiles of triplicate experiments with anchor at the conserved *MYC* promoter CTCF binding site (hg19 chr8:128,746,370, indicated as black triangle). Preferential chromatin loops ("peaks") are identified using the peakC R package and are shown in red for untreated HCT116-RAD21-mAC cells (left) and after 120 min auxin treatment (1uM 5-Ph-IAA, right). **B-C.** Natural decay of bioluminescence signal in HCT116 cells. Time-course biosensing results measured for 85 min in (**B**) untreated HCT116 cells and (**C**) HCT116 cells treated with auxin (1uM 5-Ph-IAA). Cells were co-transfected with plasmids expressing biosensor components and two different *MYC* promoter- super enhancer sgRNA pairs to measure natural decay of NanoLuc luciferase luminescence at the -0.53Mb chromatin loop. A condition where no gRNA pairs were co-transfected is shown for comparison. Luminescence was measured (n=8) every 5 min for 85 min for each transfection condition.
