# Supplementary material for: A split luciferase biosensing platform for detection and imaging of chromatin loops in individual live cells
Source: Nucleic Acids Res. 2025 Dec 3;53(22):gkaf1324. doi: 10.1093/nar/gkaf1324 (PMC12673846; doi:10.1093/nar/gkaf1324)
Supplement: gkaf1324_Supplemental_Files [file gkaf1324_supplemental_files.zip › Supplementary materials 5-1-25.pdf]

A Split Luciferase Biosensing Platform for Detection and Imaging of Chromatin Loops in Individual Live Cells

Supplementary Materials

Supplementary Note 1. Modeling the structure of cohesin and CTCF homodimerization-mediated loop anchors

Supplementary Note 2. Creation of Split luciferase dSaCas9 Fusion Proteins

Supplementary Note 3. Creation of d*Sp*Cas9 sgRNA plasmids and d*Sa*Cas9 sgRNA cassettes

Supplementary Note 4. Non-repetitive *MUC4* sgRNAs for d*Sp*Cas9 and d*Sa*Cas9

Supplementary Note 5. HCT116 and K562 *MYC* TAD Boundary and *MYC* Promoter-Super Enhancer Loop Biosensing sgRNA Designs

Supplementary Note 6. 4C-seq viewpoint and primer sequences

Supplementary Note 1. Modeling the structure of cohesin and CTCF homodimerization-mediated loop anchors

To better understand the feasibility of designing a biosensor for chromatin loops, we modeled the structure of a chromatin loop anchored by cohesin and a CTCF homodimer. Although to date, a native chromatin loop structure has not been captured *in situ* using X-ray crystallography or nuclear magnetic resonance (NMR) spectroscopy as it is a highly dynamic and often transient assembly involving multiple protein complexes, reasonable models can be made for such a structure for the purposes of biosensor design via combination of known crystal and NMR structures and a full predicted structure from AlphaFold. Starting with the published crystal structure of ZnFs 4-10 of CTCF bound to 28-mer DNA[1] (Supplementary Fig. S3a) and the discovery of an N- terminal segment of CTCF that directly interacts with the SA2-SCC1 subcomplex of cohesin and is specifically required for cohesin positioning at CTCF binding sites and for the formation of CTCF-anchored loops[2] (Supplementary Fig. S3b), we began to model the structure of a cohesin and CTCF homodimerization-mediated chromatin loop anchor. From the SA2-SCC1-CTCF complex structure, a critical stretch of amino acids from positions 222-232 in CTCF—particularly Y226 and F228—associate closely with several amino acids in cohesin subunits SCC1 and SA2, including S334, I337, R338, and L341 of SCC1 and Y297, R298, and W334 of SA2[2]. Previous literature showed that SA2 contains an 86-amino-acid motif termed the stromalin conservative domain[3,4] or conserved essential surface (CES)[5,6] which coincides with the CTCF binding pocket. Thus, we needed to align this stretch of amino acids in the SA2-SCC1-CTCF structure (CES) with the correct stretch of amino acids in the full CTCF structure, then further align this hybrid structure to the structure of ZnFs 4-10 bound to DNA. We reasoned this sequence of alignments would allow us to accurately position the ZnF domains of CTCF and bound DNA in relation to the cohesin subunit structure. As the N-terminus of CTCF was missing in the crystal structure of ZnFs 4-10 and the SA2-SCC1-CTCF complex but the idea that it is an unstructured loop domain is thoroughly supported by the literature[1,2,7], we used a full CTCF structure predicted by AlphaFold to locate the N-terminal amino acids in CTCF that interact with cohesin and create a more accurate molecular model of the positioning of CTCF relative to cohesin subunits and to bound DNA. AlphaFold predicted the N-terminal and C-terminal domains missing from all published structures to be unorganized loop domains, consistent with the literature (Supplementary Fig. S3c). The absence of the N- and C-terminal loop domains in published structures of CTCF could have occurred because the innate flexibility in proteins can often result in the absence of loops and even entire domains in structures determined by x-ray diffraction or NMR spectroscopy[8]. It has recently been shown that directional CTCF binding to cognate binding sites is achieved through insertion of ZnF 3, ZnFs 4-7, and ZnFs 9-11 into the major groove along cognate CTCF binding sites[35]. All CTCF binding sites are composed of a 19 bp core sequence that has a specific directionality and is bound by ZnFs 3- 7[9]. However, some CTCF binding sites also include an additional upstream 10 bp sequence bound by ZnFs 9- 11 separated from the core sequence by a 5-6 bp intervening sequence bound by ZnF 8[9]. With this in mind, we first aligned the structures which both contained the stretch of amino acids from positions 222-232 in the N-terminus of CTCF (Supplementary Fig. S3d) then aligned ZnFs 4-10 in this aligned structure with ZnFs 4-10 in the crystal structure where these ZnFs are bound to 28-mer DNA, removing the redundant ZnFs 4-10 and loop domains predicted by AlphaFold from the combined complex for clarity (Supplementary Fig. S3e).

Then, we created a full loop anchor model with two *cis*-interacting DNA sites. To do this, we

reconciled the remarkable fact that the cohesin complex, having a diameter of only ~50 nm[10,11] can discriminate between approach of comparatively tiny DNA-bound CTCF protein (~3-5 nm) from the N-terminal or C-terminal side during extrusion of a chromatin loop[11,12] with the well described preference for CTCF-CTCF homodimers in CTCF-anchored chromatin loops to be in the convergent orientation[13,14]. As there is substantial evidence that CTCF proteins homodimerize *in vivo*[15] and the N-termini of two homodimerizing CTCF proteins must align in loop anchors of the preferred convergent orientation[11], we first modeled the CTCF homodimer in the case where N-terminal CTCF domains align fairly closely, at ~15 Å apart (Supplementary Fig. S4, left panel). In the case where ZnF domains were aligned ~15 Å apart, the largest distance between *cis*-interacting DNA sequences was found to be ~40 Å (~4 nm) while the smallest distance was found to be ~10 Å (~1 nm). Often, side chain interactions between residues at homodimer interfaces can be <4.5 Å[16] and a large protein such as CTCF (727 amino acids) likely adopts a relatively tightly associated 3-state with dimer intermediate (3DSI) homodimerization interface[17] with the main interacting domain being the unstructured N-terminal loop domain[18,19]. Thus, we also modeled the case of a very tight dimerization interface where N-terminal CTCF domains were ~4.5 Å apart (Supplementary Fig. S4, right panel). In these tight dimerization interface models, we found that the largest distance between cis-interacting DNA sequences was ~30 Å (~3 nm) while the smallest distance was found to be ~7 Å (~0.7 nm). Thus, perhaps unsurprisingly, we found distance between *cis*-interacting DNA sequences was positively correlated with distance between homodimerized CTCF proteins in our models. However, depending on the degree to which negatively charged *cis*-interacting phosphodiester backbones are neutralized and shielded via bound CTCF ZnF domains with Zn2+ cations, CTCF-CTCF homodimers dimerizing with such tight interfaces might be somewhat destabilized by electrostatic repulsion between DNA backbones[20]. Thus, this results in some uncertainty regarding the actual expected separation for two *cis*-interacting DNA sites in a cohesin-CTCF- CTCF-anchored loop. The true distance between two *cis*-interacting DNA sequences might be less than ~7 Å or more than ~40 Å depending on the error in our models.

Currently, there are two main schools of thought regarding how cohesin extrudes loops and anchors loops with CTCF. Either cohesin subunits bind to each interacting DNA site, bridging them together, or the ring structure of cohesin encloses both interacting DNA sites. Currently, the latter idea is highly favored and supported by substantial evidence[21-24]. Therefore, while still debated, the idea that monomeric cohesin extrudes loops is currently preferred. The SMC1 and SMC3 domains of cohesin contain antiparallel coiled coil domains that allow for dimerization, creating a hinge-like structure and completing a ring structure bridged on the opposite terminus by SCC3 and SCC1/ domains[25]. Since the *cis*-interacting DNA sequences shown in the models in Supplementary Fig. S4 and S5 are stabilized via homodimerization of CTCF proteins and anchored by attachment of CTCF proteins to cohesin SA2 and SCC1/RAD21 domains, if these sequences were to be extended toward the SMC1/SMC3 hinge, they would be inserted through the cohesin ring structure starting from the attachment point of CTCF to cohesin at the SCC1/RAD21 and SA2 domains. On both sides of the loop anchor moving away from the CTCF binding sites, the distance between *cis*-interacting DNA sequences is likely to increase at a certain rate as they become unanchored by cohesin-CTCF-DNA interactions. On the side of the loop anchor closest to the insertion point of the *cis*-interacting DNA sequences to the cohesin ring, the distance between them would increase, but only to an upper bound of ~50 nm. However, if these sequences were to be extended away from the SMC1/SMC3 hinge, it is possible the distance between them would increase more quickly due to lack of constraint. Thus, we concluded that the ~50 nm cohesin ring

diameter formed an upper bound for the distance between interacting DNA sites within a reasonably short distance from the two CTCF binding sites. To date, the relationship of distance between *cis*-interacting DNA sequences and spacing from the core CTCF binding sites at loop anchor regions has not been determined. For all models, to ensure we measured distances across the loop anchor as close as possible to the more predictably anchored core CTCF binding sites and immediately adjacent sequence instead of the more unpredictably anchored extensions from the loop anchor in each direction on the DNA, we measured distances between *cis*-interacting DNA sequences across the loop anchor immediately upstream of the non-core 10 bp CTCF binding module (Supplementary Fig. S4 and S5). Thus, we established from our models that the degree of separation between CTCF monomers within the homodimer structure created a lower bound for the distance between interacting DNA sites of ~0.7-4 nm, well within the range of interaction distances at which our LgBiT-d*Sa*Cas9 and d*Sp*Cas9-SmBiT probe might operate given by results of our previous studies showing signal-to-background production at linear DNA distances of ~3.4-17 nm for single and dual dCas9 species DNA biosensors[26]. In addition, our model showed that proximal to the two CTCF binding sites, any two *cis*-interacting DNA sequences in a loop anchor are likely not separated by a distance near the cohesin ring diameter but are instead more proximal within the loop anchor structure.

<u>References for Suppl. Note 1</u>

1. Hashimoto, H. *et al*. Structural basis for the versatile and methylation-dependent binding of CTCF to DNA. *Mol. Cell* ***66***(5), 711–720.e3 (2017).
2. Li, Y. *et al*. The structural basis for cohesin-CTCF-anchored loops. *Nature* ***578***(7795), 472–476 (2020).
3. Pezzi, N. *et al*. STAG3, a novel gene encoding a protein involved in meiotic chromosome pairing and location of STAG3-related genes flanking the Williams-Beuren syndrome deletion. *FASEB J.* ***14***, 581– 592 (2000).
4. Orgil, O. *et al*. A conserved domain in the Scc3 subunit of cohesin mediates the interaction with both Mcd1 and the cohesin loader complex. *PLoS Genet.* ***11***, e1005036 (2015).
5. Hara, K. *et al*. Structure of cohesin subcomplex pinpoints direct shugoshin–Wapl antagonism in centromeric cohesion. *Nat. Struct. Mol. Biol.* ***21***, 864–870 (2014).
6. Roig, M. B. *et al*. Structure and function of cohesin's Scc3/SA regulatory subunit. *FEBS Lett.* ***588***, 3692–3702 (2014).
7. Davidson, I. F. *et al*. CTCF is a DNA-tension-dependent barrier to cohesin-mediated loop extrusion. *Nature* ***616***(7958), 822–827 (2023).
8. Petoukhov, M. V., Eady, N. A., Brown, K. A., & Svergun, D. I. Addition of missing loops and domains to protein models by x-ray solution scattering. *Biophys. J.* ***83***(6), 3113–3125 (2002).
9. Yin, M. *et al*. Molecular mechanism of directional CTCF recognition of a diverse range of genomic sites. Cell Res. ***27***(11), 1365-1377 (2017).
10. Yatskevich S, Rhodes J, Nasmyth K. Organization of chromosomal DNA by SMC complexes. *Annu. Rev. Genet.* ***53***(1), 445–482 (2019).
11. Hansen, A. CTCF as a boundary factor for cohesin-mediated loop extrusion: evidence for a multi-step mechanism. *Nucleus* ***11***(1), 132-148 (2020).
12. Nagy, G. *et al*. Motif oriented high-resolution analysis of ChIP-seq data reveals the topological order of CTCF and cohesin proteins on DNA. *BMC Genom.* ***17***(1), 637 (2016).
13. Nichols, M. H. & Corces, V. G. A CTCF code for 3D genome architecture. *Cell* ***162***(4), 703–705 (2015).

14. Tang, Z. *et al*. CTCF-mediated human 3D genome architecture reveals chromatin topology for transcription. *Cell* ***163***(7), 1611–1627 (2015).
15. Yusufzai, T.M., Tagami, H., Nakatani, Y., & Felsenfeld, G. CTCF tethers an insulator to subnuclear sites, suggesting shared insulator mechanisms across species. *Mol. Cell* ***13***, 291–298 (2004).
16. Brinda, K. V., Kannan, N., & Vishveshwara, N. Analysis of homodimeric protein interfaces by graph-spectral methods. *Protein Eng.* ***15***(4), 265-277 (2002).
17. Karthikraja, V., Suresh, A., Lulu, S., Kangueane, U., & Kangueane, P. Types of interfaces for homodimer folding and binding. *Bioinformation* ***4***(3), 101-111 (2009).
18. Fursenko D.V., Georgiev P.G., & Bonchuk A.N. Study of the N-Terminal domain homodimerization in human proteins with zinc finger clusters. *Dokl Biochem. Biophys.* ***499***(1), 257-259 (2021).
19. Bonchuk, A. *et al*. N-terminal domain of the architectural protein CTCF has similar structural organization and ability to self-association in bilaterian organisms. *Sci Rep*. ***10***(1), 2677 (2020).
20. Kornyshev, A. A., & Leikin, S. Electrostatic interaction between helical macromolecules in dense aggregates: an impetus for DNA poly- and meso-morphism. *Proc. Natl. Acad. Sci. U S A* ***95***(23), 13579– 13584 (1998).
21. Gruber, S., Haering, C.H. & Nasmyth, K. Chromosomal cohesin forms a ring. *Cell* ***112***(6): 765–77 (2003).
22. Peters, J. M., Tedeschi, A. & Schmitz, J. The cohesin complex and its roles in chromosome biology. *Genes Dev*. ***22***(22): 3089–114 (2008).
23. Kim, Y., Shi, Z., Zhang, H., Finkelstein, I. J., & Yu, H. Human cohesin compacts DNA by loop extrusion. *Science* ***366***(6471), 1345–1349 (2019).
24. Davidson, I.F. *et al*. DNA loop extrusion by human cohesin. *Science* ***366***(6471), 1338–1345 (2019).
25. Losada, A., Hirano, M., & Hirano, T. Identification of Xenopus SMC protein complexes required for sister chromatid cohesion. *Genes Dev*. ***12***(13), 1986–1997 (1998).
26. Heath, N. G., O'Geen, H., Halmai, N. B., Corn, J. E., & Segal, D. J. Imaging unique DNA sequences in individual cells using a CRISPR-Cas9-Based, split luciferase biosensor. *Front. Genome Ed.* ***4***, 867390 (2022).

Supplementary Note 2. Creation of Split luciferase dSaCas9 Fusion Proteins

Oligos for KRAB removal for cloning C-terminal dSaCas9 constructs	
F-oligo:	GACGATGACGATAAGGGTGGCGGGTCCGGCGGTGGATCCGGTACCAGCCTGAGCGGCCGG
R-oligo:	TGATGCCGATGGCCAGGCCCAGGATGTAGTTCCGCTTTGGCCGGCCGCTCAGGCTGGTAC
gBlocks used in N- and C-terminal cloning of LgBiT and SmBiT with dSaCas9 (SpCas9 constructs have previously been published)	
LgBiT-d*Sa* Cas9 (N-terminal fusion)	GACGATGACGATAAGGGTGGCGGGTCCGGCGGTGGATCCGGTACCATGGTCTTCACACTCGAAGATTTCGTTGGGGACTGGGAACAGACAGCCGCCTACAACCTGGACC<br>AAGTCCTTGAACAGGGAGGTGTGTCCAGTTTGCTGCAGAATCTCGCCGTGTCCGTAACTCCGATCCAAAGGATTGTCCGGAGCGGTGAAAATGCCCTGAAGATCGACAT<br>CCATGTCATCATCCCGTATGAAGGTCTGAGCGCCGACCAAATGGCCCAGATCGAAGAGGTGTTTAAGGTGGTGTACCCTGTGGATGATCATCACTTTAAGGTGATCCTG<br>CCCTATGGCACACTGGTAATCGACGGGGTTACGCCGAACATGCTGAACTATTTCGGACGGCCGTATGAAGGCATCGCCGTGTTCGACGGCAAAAAGATCACTGTAACAG<br>GGACCCTGTGGAACGGCAACAAAATTATCGACGAGCGCCTGATCACCCCCGACGGCTCCATGCTGTTCCGAGTAACCATCAACAGTGGTGGTAGTGGAGGTTCAGGAGG<br>ATCCGGGGGGAGCGGAGGGAGCGGCCGGCCAAAGCGGAACTACATCCTGGGCCTGGCCATCGGCATCA
SmBiT-d*Sa* Cas9 (N-terminal fusion)	GACGATGACGATAAGGGTGGCGGGTCCGGCGGTGGATCCGGTACCATGGTGACCGGCTACCGGCTGTTCGAGGAGATTCTCGGTGGTAGTGGAGGTTCAGGAGGATCCG<br>GGGGGAGCGGAGGGAGCGGCCGGCCAAAGCGGAACTACATCCTGGGCCTGGCCATCGGCATCA
d*Sa* Cas9-LgBiT (C-terminal fusion)	AGTGAAATCTAAGAAGCACCCTCAGATCATCAAAAAGGGCGCTAGCGGTGGTAGTGGAGGTTCAGGAGGATCCGGGGGGAGCGGAGGGAGCATGGTCTTCACACTCGAA<br>GATTTCGTTGGGGACTGGGAACAGACAGCCGCCTACAACCTGGACCAAGTCCTTGAACAGGGAGGTGTGTCCAGTTTGCTGCAGAATCTCGCCGTGTCCGTAACTCCGA<br>TCCAAAGGATTGTCCGGAGCGGTGAAAATGCCCTGAAGATCGACATCCATGTCATCATCCCGTATGAAGGTCTGAGCGCCGACCAAATGGCCCAGATCGAAGAGGTGTT<br>TAAGGTGGTGTACCCTGTGGATGATCATCACTTTAAGGTGATCCTGCCCTATGGCACACTGGTAATCGACGGGGTTACGCCGAACATGCTGAACTATTTCGGACGGCCG<br>TATGAAGGCATCGCCGTGTTCGACGGCAAAAAGATCACTGTAACAGGGACCCTGTGGAACGGCAACAAAATTATCGACGAGCGCCTGATCACCCCCGACGGCTCCATGC<br>TGTTCCGAGTAACCATCAACAGCGGTGGAGGCTCCGGAGGTGGATCTAAAAGGCCGGCGGCCACGAAAAAGGCCGGTCAGGCAAAAAAGAAAAAGGCGGCCGCATGAAA<br>GGGTTCGATCCCTACCGGTTAGTAATGAGTTTAAAC
d*Sa* Cas9-SmBiT (C-terminal fusion)	AGTGAAATCTAAGAAGCACCCTCAGATCATCAAAAAGGGCGCTAGCGGTGGTAGTGGAGGTTCAGGAGGATCCGGGGGGAGCGGAGGGAGCGTGACCGGCTACCGGCTG<br>TTCGAGGAGATTCTGGGTGGAGGCTCCGGAGGTGGATCTAAAAGGCCGGCGGCCACGAAAAAGGCCGGTCAGGCAAAAAAGAAAAAGGCGGCCGCATGAAAGGGTTCGA<br>TCCCTACCGGTTAGTAATGAGTTTAAAC

<u>**Final verified protein sequences**</u>

LgBiT-d*Sa*Cas9:

KEY: SV40 NLS, 3X FLAG epitope, d*Sa*Cas9, LgBiT, Nucleoplasmin NLS, P2A, variable length flexible linkers

MPKKKRKVGGSGGSDYKDHDGDYKDHDIDYKDDDDKGGGSGGGSGTMVFTLEDFVGDWEQTAAYNLDQVL
EQGGVSSLLQNLAVSVTPIQRIVRSGENALKIDIHVIIPYEGLSADQMAQIEEVFKVVYPVDDHHFKVIL
PYGTLVIDGVTPNMLNYFGRPYEGIAVFDGKKITVTGTLWNGNKIIDERLITPDGSMLFRVTINSGGSGG
SGGSGGSGGSGRPKRNYILGLAIGITSVGYGIIDYETRDVIDAGVRLFKEANVENNEGRRSKRGARRLKR
RRRHRIQRVKKLLFDYNLLTDHSELSGINPYEARVKGLSQKLSEEEFSAALLHLAKRRGVHNVNEVEEDT
GNELSTKEQISRNSKALEEKYVAELQLERLKKDGEVRGSINRFKTSDYVKEAKQLLKVQKAYHQLDQSFI
DTYIDLLETRRTYYEGPGEGSPFGWKDIKEWYEMLMGHCTYFPEELRSVKYAYNADLYNALNDLNNLVIT
RDENEKLEYYEKFQIIENVFKQKKKPTLKQIAKEILVNEEDIKGYRVTSTGKPEFTNLKVYHDIKDITAR
KEIIENAELLDQIAKILTIYQSSEDIQEELTNLNSELTQEEIEQISNLKGYTGTHNLSLKAINLILDELW
HTNDNQIAIFNRLKLVPKKVDLSQQKEIPTTLVDDFILSPVVKRSFIQSIKVINAIIKKYGLPNDIIIEL
AREKNSKDAQKMINEMQKRNRQTNERIEEIIRTTGKENAKYLIEKIKLHDMQEGKCLYSLEAIPLEDLLN
NPFNYEVDHIIPRSVSFDNSFNNKVLVKQEEASKKGNRTPFQYLSSSDSKISYETFKKHILNLAKGKGRI
SKTKKEYLLEERDINRFSVQKDFINRNLVDTRYATRGLMNLLRSYFRVNNLDVKVKSINGGFTSFLRRKW
KFKKERNKGYKHHAEDALIIANADFIFKEWKKLDKAKKVMENQMFEEKQAESMPEIETEQEYKEIFITPH
QIKHIKDFKDYKYSHRVDKKPNRELINDTLYSTRKDDKGNTLIVNNLNGLYDKDNDKLKKLINKSPEKLL
MYHHDPQTYQKLKLIMEQYGDEKNPLYKYYEETGNYLTKYSKKDNGPVIKKIKYYGNKLNAHLDITDDYP
NSRNKVVKLSLKPYRFDVYLDNGVYKFVTVKNLDVIKKENYYEVNSKCYEEAKKLKKISNQAEFIASFYN
NDLIKINGELYRVIGVNNDLLNRIEVNMIDITYREYLENMNDKRPPRIIKTIASKTQSIKKYSTDILGNL
YEVKSKKHPQIIKKGASGGGSGGGSKRPAATKKAGQAKKKKGGSGSGATNFSLLKQAGDVEENPGPAAA*

SmBiT-d*Sa*Cas9:

KEY: SV40 NLS, 3X FLAG epitope, d*Sa*Cas9, SmBiT, Nucleoplasmin NLS, variable length flexible linkers

MPKKKRKVGGSGGSDYKDHDGDYKDHDIDYKDDDDKGGGSGGGSGTMVTGYRLFEEILGGSGGSGGSGGS
GGSGRPKRNYILGLAIGITSVGYGIIDYETRDVIDAGVRLFKEANVENNEGRRSKRGARRLKRRRRHRIQ
RVKKLLFDYNLLTDHSELSGINPYEARVKGLSQKLSEEEFSAALLHLAKRRGVHNVNEVEEDTGNELSTK
EQISRNSKALEEKYVAELQLERLKKDGEVRGSINRFKTSDYVKEAKQLLKVQKAYHQLDQSFIDTYIDLL
ETRRTYYEGPGEGSPFGWKDIKEWYEMLMGHCTYFPEELRSVKYAYNADLYNALNDLNNLVITRDENEKL
EYYEKFQIIENVFKQKKKPTLKQIAKEILVNEEDIKGYRVTSTGKPEFTNLKVYHDIKDITARKEIIENA
ELLDQIAKILTIYQSSEDIQEELTNLNSELTQEEIEQISNLKGYTGTHNLSLKAINLILDELWHTNDNQI
AIFNRLKLVPKKVDLSQQKEIPTTLVDDFILSPVVKRSFIQSIKVINAIIKKYGLPNDIIIELAREKNSK
DAQKMINEMQKRNRQTNERIEEIIRTTGKENAKYLIEKIKLHDMQEGKCLYSLEAIPLEDLLNNPFNYEV
DHIIPRSVSFDNSFNNKVLVKQEEASKKGNRTPFQYLSSSDSKISYETFKKHILNLAKGKGRISKTKKEY
LLEERDINRFSVQKDFINRNLVDTRYATRGLMNLLRSYFRVNNLDVKVKSINGGFTSFLRRKWKFKKERN
KGYKHHAEDALIIANADFIFKEWKKLDKAKKVMENQMFEEKQAESMPEIETEQEYKEIFITPHQIKHIKD
FKDYKYSHRVDKKPNRELINDTLYSTRKDDKGNTLIVNNLNGLYDKDNDKLKKLINKSPEKLLMYHHDPQ
TYQKLKLIMEQYGDEKNPLYKYYEETGNYLTKYSKKDNGPVIKKIKYYGNKLNAHLDITDDYPNSRNKVV
KLSLKPYRFDVYLDNGVYKFVTVKNLDVIKKENYYEVNSKCYEEAKKLKKISNQAEFIASFYNNDLIKIN
GELYRVIGVNNDLLNRIEVNMIDITYREYLENMNDKRPPRIIKTIASKTQSIKKYSTDILGNLYEVKSKK
HPQIIKKGASGGGSGGGSKRPAATKKAGQAKKKKGGSGSGATNFSLLKQAGDVEENPGPAAA*

d*Sa*Cas9-LgBiT:

KEY: SV40 NLS, 3X FLAG epitope, d*Sa*Cas9, LgBiT, Nucleoplasmin NLS, variable length flexible linkers

MPKKKRKVGGSGGSDYKDHDGDYKDHDIDYKDDDDKGGGSGGGSGTSLSGRPKRNYILGLAIGITSVGYG
IIDYETRDVIDAGVRLFKEANVENNEGRRSKRGARRLKRRRRHRIQRVKKLLFDYNLLTDHSELSGINPY
EARVKGLSQKLSEEEFSAALLHLAKRRGVHNVNEVEEDTGNELSTKEQISRNSKALEEKYVAELQLERLK
KDGEVRGSINRFKTSDYVKEAKQLLKVQKAYHQLDQSFIDTYIDLLETRRTYYEGPGEGSPFGWKDIKEW
YEMLMGHCTYFPEELRSVKYAYNADLYNALNDLNNLVITRDENEKLEYYEKFQIIENVFKQKKKPTLKQI
AKEILVNEEDIKGYRVTSTGKPEFTNLKVYHDIKDITARKEIIENAELLDQIAKILTIYQSSEDIQEELT
NLNSELTQEEIEQISNLKGYTGTHNLSLKAINLILDELWHTNDNQIAIFNRLKLVPKKVDLSQQKEIPTT
LVDDFILSPVVKRSFIQSIKVINAIIKKYGLPNDIIIELAREKNSKDAQKMINEMQKRNRQTNERIEEII
RTTGKENAKYLIEKIKLHDMQEGKCLYSLEAIPLEDLLNNPFNYEVDHIIPRSVSFDNSFNNKVLVKQEE
ASKKGNRTPFQYLSSSDSKISYETFKKHILNLAKGKGRISKTKKEYLLEERDINRFSVQKDFINRNLVDT
RYATRGLMNLLRSYFRVNNLDVKVKSINGGFTSFLRRKWKFKKERNKGYKHHAEDALIIANADFIFKEWK
KLDKAKKVMENQMFEEKQAESMPEIETEQEYKEIFITPHQIKHIKDFKDYKYSHRVDKKPNRELINDTLY
STRKDDKGNTLIVNNLNGLYDKDNDKLKKLINKSPEKLLMYHHDPQTYQKLKLIMEQYGDEKNPLYKYYE
ETGNYLTKYSKKDNGPVIKKIKYYGNKLNAHLDITDDYPNSRNKVVKLSLKPYRFDVYLDNGVYKFVTVK
NLDVIKKENYYEVNSKCYEEAKKLKKISNQAEFIASFYNNDLIKINGELYRVIGVNNDLLNRIEVNMIDI
TYREYLENMNDKRPPRIIKTIASKTQSIKKYSTDILGNLYEVKSKKHPQIIKKGASGGSGGSGGSGGSGG
SMVFTLEDFVGDWEQTAAYNLDQVLEQGGVSSLLQNLAVSVTPIQRIVRSGENALKIDIHVIIPYEGLSA
DQMAQIEEVFKVVYPVDDHHFKVILPYGTLVIDGVTPNMLNYFGRPYEGIAVFDGKKITVTGTLWNGNKI
IDERLITPDGSMLFRVTINSGGGSGGGSKRPAATKKAGQAKKKKAAA*

d*Sa*Cas9-SmBiT:

KEY: SV40 NLS, 3X FLAG epitope, d*Sa*Cas9, SmBiT, Nucleoplasmin NLS, variable length flexible linkers

MPKKKRKVGGSGGSDYKDHDGDYKDHDIDYKDDDDKGGGSGGGSGTSLSGRPKRNYILGLAIGITSVGYG
IIDYETRDVIDAGVRLFKEANVENNEGRRSKRGARRLKRRRRHRIQRVKKLLFDYNLLTDHSELSGINPY
EARVKGLSQKLSEEEFSAALLHLAKRRGVHNVNEVEEDTGNELSTKEQISRNSKALEEKYVAELQLERLK
KDGEVRGSINRFKTSDYVKEAKQLLKVQKAYHQLDQSFIDTYIDLLETRRTYYEGPGEGSPFGWKDIKEW
YEMLMGHCTYFPEELRSVKYAYNADLYNALNDLNNLVITRDENEKLEYYEKFQIIENVFKQKKKPTLKQI
AKEILVNEEDIKGYRVTSTGKPEFTNLKVYHDIKDITARKEIIENAELLDQIAKILTIYQSSEDIQEELT
NLNSELTQEEIEQISNLKGYTGTHNLSLKAINLILDELWHTNDNQIAIFNRLKLVPKKVDLSQQKEIPTT
LVDDFILSPVVKRSFIQSIKVINAIIKKYGLPNDIIIELAREKNSKDAQKMINEMQKRNRQTNERIEEII
RTTGKENAKYLIEKIKLHDMQEGKCLYSLEAIPLEDLLNNPFNYEVDHIIPRSVSFDNSFNNKVLVKQEE
ASKKGNRTPFQYLSSSDSKISYETFKKHILNLAKGKGRISKTKKEYLLEERDINRFSVQKDFINRNLVDT
RYATRGLMNLLRSYFRVNNLDVKVKSINGGFTSFLRRKWKFKKERNKGYKHHAEDALIIANADFIFKEWK
KLDKAKKVMENQMFEEKQAESMPEIETEQEYKEIFITPHQIKHIKDFKDYKYSHRVDKKPNRELINDTLY
STRKDDKGNTLIVNNLNGLYDKDNDKLKKLINKSPEKLLMYHHDPQTYQKLKLIMEQYGDEKNPLYKYYE
ETGNYLTKYSKKDNGPVIKKIKYYGNKLNAHLDITDDYPNSRNKVVKLSLKPYRFDVYLDNGVYKFVTVK
NLDVIKKENYYEVNSKCYEEAKKLKKISNQAEFIASFYNNDLIKINGELYRVIGVNNDLLNRIEVNMIDI
TYREYLENMNDKRPPRIIKTIASKTQSIKKYSTDILGNLYEVKSKKHPQIIKKGASGGSGGSGGSGGSGG
SVTGYRLFEEILGGGSGGGSKRPAATKKAGQAKKKKAAA*

Supplementary Note 3. Creation of d*Sp*Cas9 sgRNA plasmids and d*Sa*Cas9 sgRNA cassettes

Oligos for d*Sp*Cas9 sgRNA cloning:

```
Insert_F: TTTCTTGGCTTTATATATCTTGTGGAAAGGACGAAACACCGNNNNNNNNNNNNNNNNNNN
Insert_R: GACTAGCCTTATTTTAACTTGCTATTTCTAGCTCTAAAACNNNNNNNNNNNNNNNNNNNC
```

Target protospacer sequence is denoted in green in F oligo and the reverse complement sequence is red in R oligo

Design notes for self-contained dSaCas9 gRNA gBlocks:

d*Sa*Cas9 tracrRNA

GUUUUAGUACUCUGGAAACAGAAUCUACUAAAACAAGGCAAAAUGCCGUGUUUAUCUCGUCAACUUGUUG GCGAGAUUUUUU

Sense DNA strand for synthesis:

GTTTTAGTACTCTGGAAACAGAATCTACTAAAACAAGGCAAAATGCCGTGTTTATCTCGTCAACTTGTTG GCGAGATTTTTT

Final d*Sa*Cas9 sgRNA expression cassette gBlock design:

KEY: U6 promoter, 5' G for enhanced U6 transcription, 21 nt d*Sa*Cas9 sgRNA target sequence, dSaCas9 sgRNA scaffold, termination signal

TGTACAAAAAAGCAGGCTTTAAAGGAACCAATTCAGTCGACTGGATCCGGTACCAAGGTCGGGCAGGAAG
AGGGCCTATTTCCCATGATTCCTTCATATTTGCATATACGATACAAGGCTGTTAGAGAGATAATTAGAAT
TAATTTGACTGTAAACACAAAGATATTAGTACAAAATACGTGACGTAGAAAGTAATAATTTCTTGGGTAG
TTTGCAGTTTTAAAATTATGTTTTAAAATGGACTATCATATGCTTACCGTAACTTGAAAGTATTTCGATT
TCTTGGCTTTATATATCTTGTGGAAAGGACGAAACACCGNNNNNNNNNNNNNNNNNNNNNGTTTTAGTAC
TCTGGAAACAGAATCTACTAAAACAAGGCAAAATGCCGTGTTTATCTCGTCAACTTGTTGGCGAGATTTT
TTCTAGACCCAGCTTTCTTGTACAAAGTTGGCATTA

Note: Primer set for gBlock amplification with Q5 Hot Start High-Fidelity Polymerase is underlined

Supplementary Note 4. Non-repetitive *MUC4* sgRNAs for d*Sp*Cas9 and d*Sa*Cas9 (Fig.1, Fig.S1)

**d*Sp*Cas9 and d*Sa*Cas9 target sites in non-repetitive MUC4 intron 1 [hg19_chr3:195534754-195538017]**			
***Sp*Cas9 gRNAs targeting non-repetivitve sites in *MUC4* intron**			
MUC4 SpgRNA1	TGAGTCCTTTGCGTCGCTAA**GGG**	tandem [10 bp] with SpgRNA1-1	
MUC4 SpgRNA1-1	AGCTGGGCCAGGAGAGGAGA**TGG**		
MUC4 SpgRNA2	AGGCTAAGAACAGTCGCCGA**GGG**	tandem [17 bp] with SpgRNA2-1	
MUC4 SpgRNA2-1	CTCCTGCCCTGCCTCTCAGC**AGG**		
MUC4 SpgRNA3	TGGTGTCTACTGGTCGCCCC**AGG**	tandem [15 bp] with SpgRNA3-1	
MUC4 SpgRNA3-1	CCAGGCAGGAATGACTCAGA**AGG**		
MUC4 SpgRNA4	GGGGCTCGTAGCCCCGGCAT**TGG**	tandem [4 bp] with SpgRNA4-1	
MUC4 SpgRNA4-1	GTAGCCCCGGCATTGGCCTT**GGG**		
MUC4 SpgRNA5	GGGGAAGGGGCTACTACGTA**GGG**	tandem [8 bp] with SpgRNA5-1	
MUC4 SpgRNA5-1	TACTACGTAGGGTTGTCATG**AGG**		
MUC4 SpgRNA6	TGGATCCTTCCAGACATCGC**CGG**	inverted with SpgRNA 6-1	
MUC4 SpgRNA6-1	CAGAGGGCCAGAGAGCAGCC**CGG**		
MUC4 SpgRNA7	GGCTTGGTGTATTCAGAATG**TGG**	tandem [3 bp] with SpgRNA7-1	
MUC4 SpgRNA7-1	GCTGCATGAACGGACCCCGA**GGG**		
MUC4 SpgRNA8	GGGGCTACGAGCCCCAGGCA**AGG**	inverted 5 bp with SaCas9 gRNA4	
MUC4 SpgRNA9	ATGCCGGGGCTACGAGCCCC**AGG**	inverted 10 bp with SaCas9 gRNA4	
MUC4 SpgRNA10	GAAGGACTGTTGGTGTGCAA**GGG**	inverted 13 bp with SaCas9 gRNA1	
MUC4 SpgRNA11	GCTCTAGGGCCATGGCCCTC**GGG**	inverted 22 bp with SaCas9 gRNA7	
MUC4 SpgRNA12	GAGGAGCCCAAGGCCAATGC**CGG**	inverted 26 bp with SaCas9 gRNA4	
MUC4 SpgRNA13	GACATCGCCGGGCTGCTCTC**TGG**	inverted 31 bp with SaCas9 gRNA6	
MUC4 SpgRNA14	CTGGAGCGAGCCCCTGCTCT**AGG**	inverted 37 bp with SaCas9 gRNA7	
MUC4 SpgRNA15	GCCAAGGGCGTCGCTGATGC**TGG**	everted 12 bp with SaCas9 gRNA5	
MUC4 SpgRNA16	TCATTGCGGCAGCTCCTTTG**TGG**	everted 17 bp with SaCas9 gRNA3	
MUC4 SpgRNA17	TAAGGGGACCAAGTGGAGCT**GGG**	everted 21 bp with SaCas9 gRNA1	
MUC4 SpgRNA18	CAGAAACACAGAATAAAACT**TGG**	everted 24 bp with SaCas9 gRNA2	
MUC4 SpgRNA19	AAGTGGAGCTGGGCCAGGAG**AGG**	everted 31 bp with SaCas9 gRNA1	
MUC4 SpgRNA20	CTGTGTTTTTCCTCTGGCCT**GGG**	everted 45 bp with SaCas9 gRNA3	
***dSa*Cas9 gRNAs targeting non-repetivitve sites in *MUC4* intron**			
MUC4 SagRNA1	TCCACGACATGCCTAGCTTCT**TCGGGT**	everted [4 bp] with dSpCas9 g1	everted [37 bp] with dSpCas9 g1-1
MUC4 SagRNA2	TCTGGGGTCCAGAGTTCAAGC**TGGGGT**	tandem [31 bp] with dSpCas9 g2	tandem [10 bp] with dSpCas9 g2-1
MUC4 SagRNA3	AGCCCTGGCTCTCAAGGGTTA**AAGGAT**	tandem [46 bp] with dSpCas9 g3	tandem [64 bp] with dSpCas9 g3-1
MUC4 SagRNA4	CTCGGGTTTAAAAGCCTCCAT**TTGGGT**	tandem [13 bp] dSpCas9 g4	tandem [20 bp] with dSpCas9 g4-1
MUC4 SagRNA5	ACAGGCAACGCCTTTTGGCTC**TGGAGT**	tandem [31 bp] with dSpCas9 g5	tandem [40 bp] with dSpCas9 g5-1
MUC4 SagRNA6	ATCTGAGGGGAAAGACAGAGG**GAGAAT**	inverted [44 bp] with dSpCas9 g6	tandem [24 bp] with dSpCas9 g6-1
MUC4 SagRNA7	CAAGGGCTGGCTTGGTGTATT**CAGAAT**	tandem [7 bp] with dSpCas9 g7	tandem [1 bp] with dSpCas9 g7-1

Supplementary Note 5. HCT116 and K562 *MYC* TAD Boundary and *MYC* Promoter-Super Enhancer Loop Biosensing sgRNA Designs

**Control gRNA pair to non interacting target sites**	
PALB2 dSpCas9 gRNA4	CACACGAGATTATACACATC**AGG**
MYC dSaCas9 gRNA d	GGGGAGCAACCAATCGCTATG**CTGGAT**

<u>Region1:</u> Conserved CTCF site upstream of *MYC* promoter
(hg19 chr8:128746351-128746370)

Region 1 dSpCas9 gRNAs [Fig.2C, 2D, 2E]	
MYC prom SpgRNA a	TGGAGGAAAAAGTGGTTCAG**AGG**
MYC prom SpgRNA b	TGCAGAAGGTCCGAAGAAAG**AGG**
MYC prom SpgRNA c	TGTCAACGAGGGCGGGGGTC**AGG**
MYC prom SpgRNA d	TCTGGGCAGCACCCGCGTTC**AGG**
MYC prom SpgRNA e	GGAGCAACCAATCGCTATGC**TGG**
MYC prom SpgRNA f	CACTGCACAATTCAGCTTTA**AGG**
Region 1 - dSaCas9 gRNAs	
MYC prom SagRNA a	TTTTCACCATCTCTTATGCGG**TTGAAT**
MYC prom SagRNA b	CTGCAGAAGGTCCGAAGAAAG**AGGAGT**
MYC prom SagRNA c	CGCCTGGATGTCAACGAGGGC**GGGGGT**
MYC prom SagRNA d	GGGGAGCAACCAATCGCTATG**CTGGAT**
MYC prom SagRNA e	AGCTCAGCGTTCAAGTGTTAA**GTGAAT**
MYC prom SagRNA f	ATAGCATGTACGCTGTTCAAG**ATGGGT**

<u>Region 2:</u> HCT116 super enhancer 0.53 Mb upstream of *MYC* promoter CTCF
(hg19 chr8:128220222-128220241)

Region 2 - dSpCas9 gRNAs [Fig.2C]	
SE -0.53Mb SpgRNA 1	GACACAAAATTACTGTTGAA**GGG**
SE -0.53Mb SpgRNA 2	AGTTGCTACAATCACTATAT**TGG**
SE -0.53Mb SpgRNA 3	catagtgcttagAATTGAGG**GGG**
SE -0.53Mb SpgRNA 4	ccccaaaccatcccagtcca**cgg**
SE -0.53Mb SpgRNA 5	cttccactgattctacatta**tgg**
SE -0.53Mb SpgRNA 6	AGTTGCAGGAAACAAGCTCA**GGG**

<u>Region 3:</u> HCT116 E7 enhancer 0.33 Mb upstream of *MYC* promoter CTCF
(hg19 chr8:128413089-128413107)

Region 3 - dSpCas9 gRNAs [Fig.S6]	
E7 0.33Mb SpgRNA 1	TGGTTTCCTCTTAGCTCTTG**GGG**
E7 0.33Mb SpgRNA 2	TGATTTTTCCAAATTCAAAT**AGG**
E7 0.33Mb SpgRNA 3	TGGAATCATGCAATAAGAGC**AGG**
E7 0.33Mb SpgRNA 4	GTGGAGCTGGAAGGGAAATC**AGG**
E7 0.33Mb SpgRNA 5	CTGAGCTCAAAGGACGATGA**GGG**
E7 0.33Mb SpgRNA 6	TATTTTATTTTATGTGGGGG**AGG**

<u>Region 4:</u> K562 super enhancer 1.85 Mb downstream of *MYC* promoter CTCF
(hg19 chr8:130599463-130599481)

Region 4 - dSpCas9 gRNAs [Fig.2E]	
SE 1.85 Mb SpgRNA 1	CCTCAAAATGCAAAGCATCC**TGG**
SE 1.85 Mb SpgRNA 2	TCCTTGGGTCACTGTGGGGA**TGG**
SE 1.85 Mb SpgRNA 3	GATTGTCTTCACCAGTTGGG**AGG**
SE 1.85 Mb SpgRNA 4	GTTCATCTCAAACTCTCTTG**AGG**
SE 1.85 Mb SpgRNA 5	TCTGCTCTACTTAGCTATTG**AGG**
SE 1.85 Mb SpgRNA 6	TGTTTCAAATCACCTGTTAG**AGG**

<u>Region 5:</u> Super enhancer 8.2 kb upstream of *MYC* promoter CTCF
(hg19 chr8:128738136-128738154)

Region 5 - dSaCas9 gRNAs [Fig.2D]	
SE -8.2 kb SpgRNA 1	GGCAAATGATGACCCCTTAG**TGG**
SE -8.2 kb SpgRNA 2	TGGTAACAGGAACTGGGCTA**GGG**
SE -8.2 kb SpgRNA 3	AATCCTATCTGAATTGAAGA**AGG**
SE -8.2 kb SpgRNA 4	TCGTTGGAGCAAGGGTGACG**AGG**
SE -8.2 kb SpgRNA 5	GAGCTGGGCCCCAACATCGT**TGG**
SE -8.2 kb SpgRNA 6	CTTCGGTTCCATCAATGGGT**AGG**

<u>Designs for *MYC* TAD Boundary Region Loop Anchors</u>

Two strong CTCF-CTCF interactions for same TAD boundary (hg19)

1. chr8: 127881391-130694709 (ChIA-PET score: 300)
2. chr8: 127888565-130700261 (ChIA-PET score: 200)

<u>Region 6:</u> Left TAD boundary (LB) region for *MYC* insulated neighborhood

Top peak CTCF sites (hg19):

1. chr8:127881798-127881816
2. chr8:127888,996-127889,014

<u>Region 7:</u> Right TAD boundary (RB) region for *MYC* insulated neighborhood

Top peak CTCF sites (hg19):

1. chr8: 130694449-130694467
2. chr8: 130699821-130699839

Region 6 (LB) SagRNAs - CTCF peak 1 [Fig.2B]	
LB CTCF1 SagRNA 1	CCTAATGCACAGGCAGGCCCT**GGGGGT**
LB CTCF1 SagRNA 2	TTCCCACTTCCACCTTGGTGA**GGGAAT**
LB CTCF1 SagRNA 3	ATGAATGACTTGCGTGATCTG**CAGAGT**
LB CTCF1 SagRNA 4	ATCATTCACTTAGTAGGCACT**ATGAAT**
Region 6 (LB) SagRNAs - CTCF peak 2 [Fig.S5]	
LB CTCF2 SagRNA 1	TTTTTACTATCAAACCCTTGT**TGGGAT**
LB CTCF2 SagRNA 2	GGCGCCCCTGGCTTTAAAACT**GGGGAT**
LB CTCF2 SagRNA 3	CAGTTTTAAAGCCAGGGGCGC**CGGGGT**
LB CTCF2 SagRNA 4	TCTTTCTAGAAAAATCCCAAC**AAGGGT**
Region 7 (RB) SpgRNAs - CTCF peak 1 [Fig.2B]	
RB CTCF1 SpgRNA a	CACAAAGGGGCAAAGTTTAA**AGG**
RB CTCF1 SpgRNA b	GCTCACAGATGAGTAGATTC**AGG**
RB CTCF1 SpgRNA c	GACAATTCAGTCTATTGCTA**AGG**
RB CTCF1 SpgRNA d	GAAGTCACTCTGCTAGCCCA**CGG**
Region 7 (RB) SpgRNAs - CTCF peak 2 [Fig.S5]	
RB CTCF2 SpgRNA a	ATAAGCTATCGACCCTATAA**AGG**
RB CTCF2 SpgRNA b	TATGTCCGTGGACCTTTATA**GGG**
RB CTCF2 SpgRNA c	TGCCCTAGAAGTCCTGGTCA**AGG**
RB CTCF2 SpgRNA d	TTGATTCTGAGACCTTGACC**AGG**
RB CTCF2 SpgRNA e	TGCCTGGTCCACATACTCCC**TGG**
RB CTCF2 SpgRNA f	GTCCAGGGAGTATGTGGACC**AGG**
RB CTCF2 SpgRNA g	TACCTCCATGGACTAGACCT**GGG**
RB CTCF2 SpgRNA h	ACTTAAAGATGAGAAAGCCC**AGG**

SupplementaryNote 6. 4C-seq viewpoint and primer sequences

4C viewpoint fragment upstream of *MYC* promoter (283 bp):

GATCTCTGCTGCCAGTAGAGGGCACACTTACTTTACTTTCGCAAACCTGAACGCGGGTGCTGCCCAGAGAGGGGGCG
GAGGGAAAGACGCTTTGCAGCAAAATCCAGCATAGCGATTGGTTGCTCCCCGCGTTTGCGGCAAAGGCCTGGAGGCA
GGAGTAATTTGCAATCCTTAAAGCTGAATTGTGCAGTGCATCGGATTTGGAAGCTACTATATTCACTTAACACTTGA
ACGCTGAGCTGCAAACTCAACGGGTAATAACCCATCTTGAACAGCGTACATG

Creating fragment sequence for primer design using Primer3:

AGGCCTGGAGGCAGGAGTAATTTGCAATCCTTAAAGCTGAATTGTGCAGTGCATCGGATTTGGAAGCTACTATATTC
ACTTAACACTTGAACGCTGAGCTGCAAACTCAACGGGTAATAACCCATCTTGAACAGCGTACATGGATCTCTGCTGC
CAGTAGAGGGCACACTTACTTTACTTTCGCAAACCTGAACGCGGGTGCTGCCCAGAGAGGGGGCGGAGGGAAAGACG
CTTTGCAGCAAAATCCAGCATAGCGATTGGTTGCTCCCCGCGTTTGCGGCAA

Oligos for 4C-seq experiment	
**Viewpint primers**	
FP (non-reading primer—NlaIII)	GCAAACTCAACGGGTAATAA
RP (reading primer—DpnII)	GTAAGTGTGCCCTCTACTGG
**2-step library prep primer sets**	
**1st round**	
VP_RP_readp (1st_r)	TACACGACGCTCTTCCGATCTGTAAGTGTGCCCTCTACTGG
VP_FP_nonreadp (1st_r)	ACTGGAGTTCAGACGTGTGCTGCAAACTCAACGGGTAATAA
VP_FP_nonreadp + 10 bp spacer (1st_r)	ACTGGAGTTCAGACGTGTGCTCTTCCGATCTGCAAACTCAACGGGTAATAA
**2nd round**	
Universal FP (2nd_r)	AATGATACGGCGACCACCGAGATCTACACTCTTTCCCTACACGACGCTCTTCCGATCT
RP_1 (2nd_r)	CAAGCAGAAGACGGCATACGAGAT**CTTTTG**GTGACTGGAGTTCAGACGTGTGCT
RP_2 (2nd_r)	CAAGCAGAAGACGGCATACGAGAT**TAGTTG**GTGACTGGAGTTCAGACGTGTGCT
RP_3 (2nd_r)	CAAGCAGAAGACGGCATACGAGAT**CCGGTG**GTGACTGGAGTTCAGACGTGTGCT
RP_4 (2nd_r)	CAAGCAGAAGACGGCATACGAGAT**ATCGTG**GTGACTGGAGTTCAGACGTGTGCT
RP_5 (2nd_r)	CAAGCAGAAGACGGCATACGAGAT**TGAGTG**GTGACTGGAGTTCAGACGTGTGCT
RP_6 (2nd_r)	CAAGCAGAAGACGGCATACGAGAT**CGCCTG**GTGACTGGAGTTCAGACGTGTGCT
RP_7 (2nd_r)	CAAGCAGAAGACGGCATACGAGAT**GCCATG**GTGACTGGAGTTCAGACGTGTGCT
RP_8 (2nd_r)	CAAGCAGAAGACGGCATACGAGAT**AAAATG**GTGACTGGAGTTCAGACGTGTGCT
RP_9 (2nd_r)	CAAGCAGAAGACGGCATACGAGAT**TGTTGG**GTGACTGGAGTTCAGACGTGTGCT
RP_10 (2nd_r)	CAAGCAGAAGACGGCATACGAGAT**ATTCCG**GTGACTGGAGTTCAGACGTGTGCT
RP_11 (2nd_r)	CAAGCAGAAGACGGCATACGAGAT**AGCTAG**GTGACTGGAGTTCAGACGTGTGCT
RP_12 (2nd_r)	CAAGCAGAAGACGGCATACGAGAT**GTATAG**GTGACTGGAGTTCAGACGTGTGCT
RP_13 (2nd_r)	CAAGCAGAAGACGGCATACGAGAT**TCTGAG**GTGACTGGAGTTCAGACGTGTGCT
RP_14 (2nd_r)	CAAGCAGAAGACGGCATACGAGAT**GTCGTC**GTGACTGGAGTTCAGACGTGTGCT
RP_15 (2nd_r)	CAAGCAGAAGACGGCATACGAGAT**CGATTA**GTGACTGGAGTTCAGACGTGTGCT
Note: Indexes for RP 1-15 in this set equals indexes for RP 28-42 in Illumina RNA PCR set	
